# Epigenetic Transgenerational Actions of Vinclozolin on Promoter Regions of the Sperm Epigenome

**DOI:** 10.1371/journal.pone.0013100

**Published:** 2010-09-30

**Authors:** Carlos Guerrero-Bosagna, Matthew Settles, Ben Lucker, Michael K. Skinner

**Affiliations:** Center for Reproductive Biology, School of Biological Sciences, Washington State University, Pullman, Washington, United States of America; Istituto Dermopatico dell'Immacolata, Italy

## Abstract

Previous observations have demonstrated that embryonic exposure to the endocrine disruptor vinclozolin during gonadal sex determination promotes transgenerational adult onset disease such as male infertility, kidney disease, prostate disease, immune abnormalities and tumor development. The current study investigates genome-wide promoter DNA methylation alterations in the sperm of F3 generation rats whose F0 generation mother was exposed to vinclozolin. A methylated DNA immunoprecipitation with methyl-cytosine antibody followed by a promoter tilling microarray (MeDIP-Chip) procedure was used to identify 52 different regions with statistically significant altered methylation in the sperm promoter epigenome. Mass spectrometry bisulfite analysis was used to map the CpG DNA methylation and 16 differential DNA methylation regions were confirmed, while the remainder could not be analyzed due to bisulfite technical limitations. Analysis of these validated regions identified a consensus DNA sequence (motif) that associated with 75% of the promoters. Interestingly, only 16.8% of a random set of 125 promoters contained this motif. One candidate promoter (Fam111a) was found to be due to a copy number variation (CNV) and not a methylation change, suggesting initial alterations in the germline epigenome may promote genetic abnormalities such as induced CNV in later generations. This study identifies differential DNA methylation sites in promoter regions three generations after the initial exposure and identifies common genome features present in these regions. In addition to primary epimutations, a potential indirect genetic abnormality was identified, and both are postulated to be involved in the epigenetic transgenerational inheritance observed. This study confirms that an environmental agent has the ability to induce epigenetic transgenerational changes in the sperm epigenome.

## Introduction

Epigenetic changes derived from exposure to endocrine disruptors have been described in several tissues and organisms [1], [2], [3], [4], [5], [6], [7]. These endocrine disruptor induced epigenetic changes may have a wide range of phenotypic consequences with implications from disease etiology [8], [9] to evolution [7], [10], [11]. Disease conditions affected include cancers [12], [13], reproductive defects [1], [14], [15], [16] and obesity [6], [17]. Recent studies have suggested disease associated with exposure to either endocrine disruptors [1], [14], [18], [19], [20], [21] or nutrient restriction [6], [22] can become transgenerationally transmitted. In particular, exposures during embryonic gonadal development and sex determination are capable of inducing adult onset disease states that can be perpetuated across multiple generations [1], [9]. The first example involved exposure to vinclozolin, a fungicide commonly used in agriculture and known to be an anti-androgenic endocrine disrupting compound [23]. In adult male rats younger than 120 days of age that are derived from vinclozolin-exposed ancestors, the main disease phenotype observed is a spermatogenic cell defect in the testis [1], [19]. Additional transgenerational disease develops as animals age (6–14 months), including increased frequencies of tumors, prostate disease, kidney disease, immune abnormalities and other defects in spermatogenesis [14]. Transgenerational disease has also been seen in females as a consequence of vinclozolin treatment. These diseases include uterine hemorrhage and/or anemia late in pregnancy [18]. Changes in behavior and learning capacity have also been observed following vinlozolin exposure [10], [24], [25], including transgenerationally transmitted changes in mate preference [10] and anxiety behavior [25]. These transgenerational epigenetic phenotypes appear to be part of a genome-wide effect of vinclozolin treatment during germ line development. Evidence for this is that the embryonic testis transcriptome is substantially altered in males from the F1 through the F3 generations, after F0 generation maternal exposure to vinclozolin [26].

A previous report has shown that these vinclozolin-induced transgenerational effects correlate with DNA methylation [1]. DNA methylation refers to the addition of a methyl group to CG dinucleotides (CpGs) [27], which through interactions with other epigenetic systems [28] and environmental signals [7], [29] can regulate gene expression without changes in DNA sequences. Importantly, these transgenerational epigenetic effects are triggered during a window of exposure in which the germ line epigenome is developing, between embryonic days 8 to 14 (E8–E14 in the rat). The comparable period in the human is between 6 weeks and mid gestation. During this developmental period, the germ line is undergoing major reprogramming in its global DNA methylation status [29], [30], [31]. Prior to sex determination, while primordial germ cells migrate down the genital ridge towards the developing gonad, they undergo an important reduction in global DNA methylation, becoming demethylated around the time of entry into the gonads [32]. Allelic differences in DNA methylation, which is characteristic of imprinted genes, are defined during this developmental period of the germ line [33]. Therefore, external agents capable of permanently altering the germ line epigenome during this critical period of establishment of DNA methylation marks and reprogramming can persist transgenerationally [1], [9].

F3 generation epigenetic changes in DNA methylation induced by vinclozolin were previously assessed with the use of methylation-sensitive restriction enzyme digestion and bisulfite sequencing [1]. These techniques are reliable for assessing DNA methylation status at an individual gene scale, but have limitations in terms of assessing genome-wide methylation [34]. Methylated DNA immuno-precipitation (MeDIP) followed by tilling array analysis (MeDIP-Chip) is one of the tools that allows for a genome-wide approach. The procedure is based on enriching methylated DNA in a sample using immuno-precipitation with an antibody for methylated cytosine, followed by tiling microarray chip hybridizations [35]. This method has been used to map the methylome in *Arabidopsis thaliana* [36], human breast cancer metastasis [37] and the human Major Histocompatibility Complex [38]. Other studies have compared genome-wide methylation changes derived from two experimental conditions, for example assessing methylation in cancer cells compared to controls [34], [39]. However, few studies use genome-wide methylation approaches to evaluate whole organism exposures to environmental compounds. Examples exist for exposure to cocaine and BPA [40], [41].

The present study shows promoter genome-wide DNA methylation changes in the germ line of F3 generation rats whose F0 generation mothers were exposed to the endocrine disruptor vinclozolin. This study confirms with more recent technology our previous findings that an endocrine disrupting agent (vinclozolin) has the ability to induce transgenerational epigenetic modifications in the male germ line. This study identifies several promoter regions that have altered DNA methylation status three generations after the initial exposure and identifies common genomic features present in these regions. In addition to a potential common consensus motif among the regions that presented a transgenerational change in methylation, an alteration in copy number variation (CNV) was identified.

## Results

### Transgenerational Genome-wide Promoter Alterations

**Figure 1:**
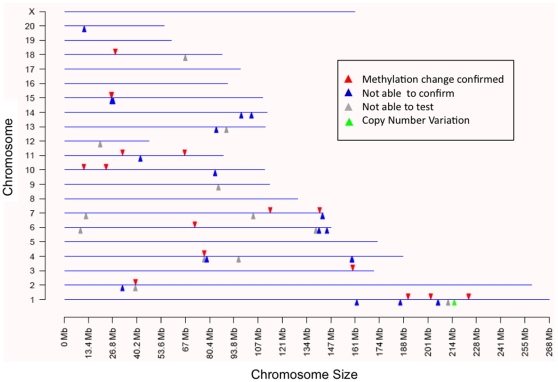
Chromosomal distribution of the transgenerational change in DNA methylation in promoter regions identified by MeDIP followed by comparative chip hybridizations. The red arrow indicates regions with confirmed methylation change through mass spectrometry. Blue arrows indicate regions in which change was not able to be confirmed, either because of insufficient CpG site measurement through mass spectrometry or not changed. Grey arrows indicate regions where primers could not be designed to test them. Green arrow indicates a transgenerational copy number variation (CNV) event in Fam111a.

**Figure 2:**
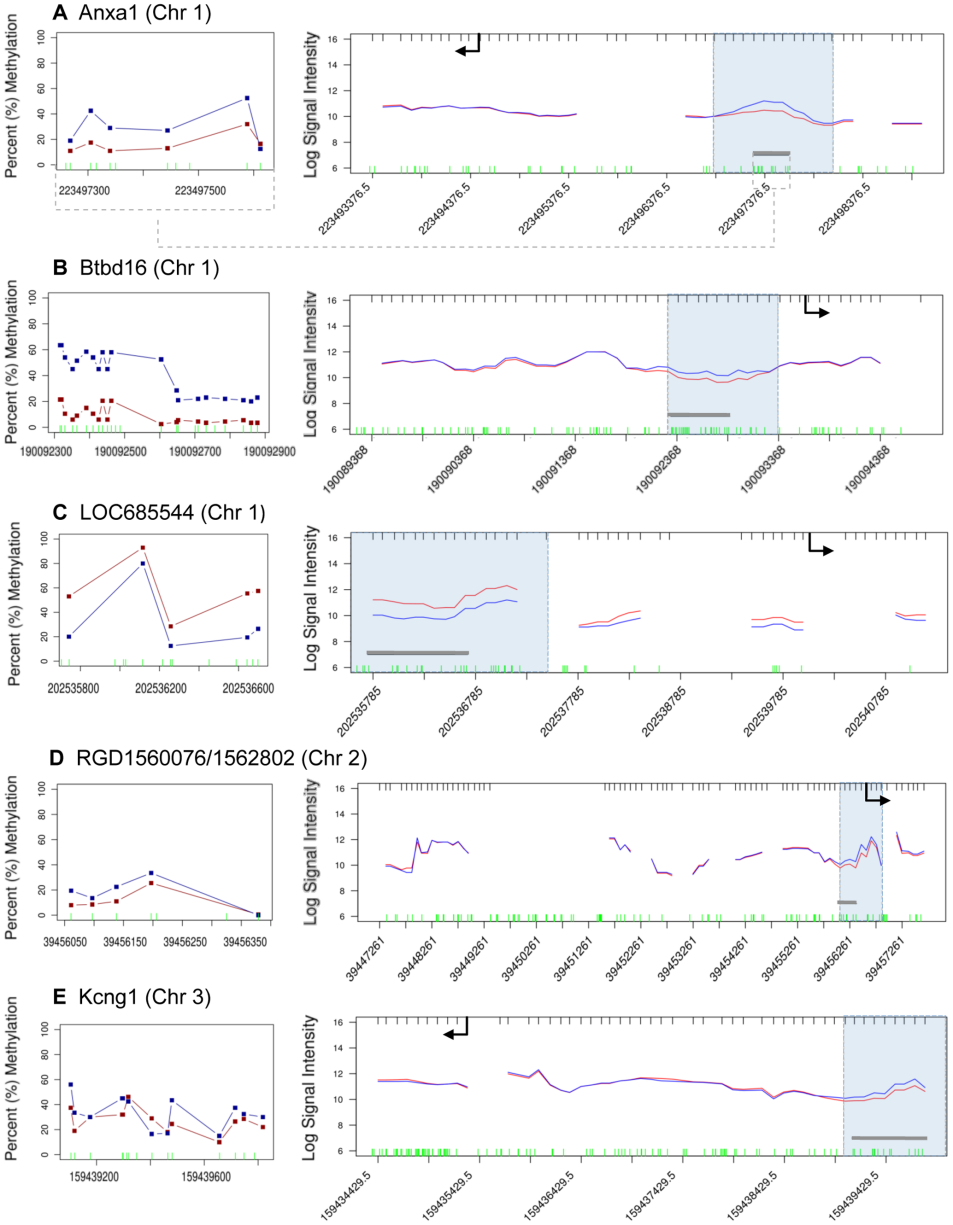
Comparison of the methylation signal in regions where transgenerational methylation change is confirmed between vinclozolin and control. Analysis of methylation through MeDIP followed by comparative hybridization is graph on right with genome location and log signal intensity presented with shaded area being the differential methylation region. Heavy red line indicates control and blue line indicates vinclozolin and arrow indicates transcriptional start site and direction. The graph on left is the bisulfite mass spectrometry analysis of CpG sites within the bar in shaded area of right graph indicated with percent methylation data presented for each gene **(a-n)**. Horizontal axis shows chromosomal localizations. Inset legend presented in Figure 4.

**Figure 3:**
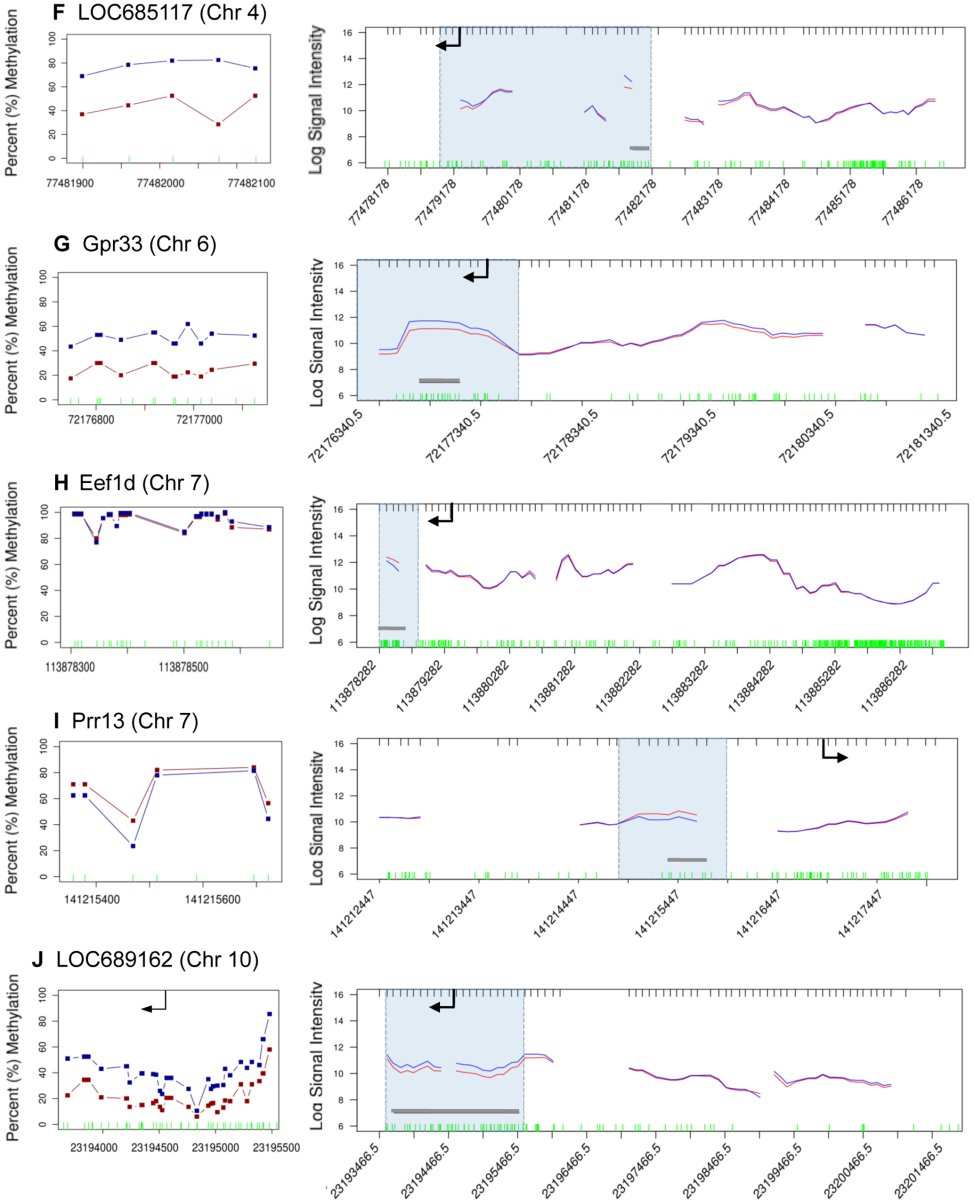
Refer to **Figure 2** Legend.

**Figure 4:**
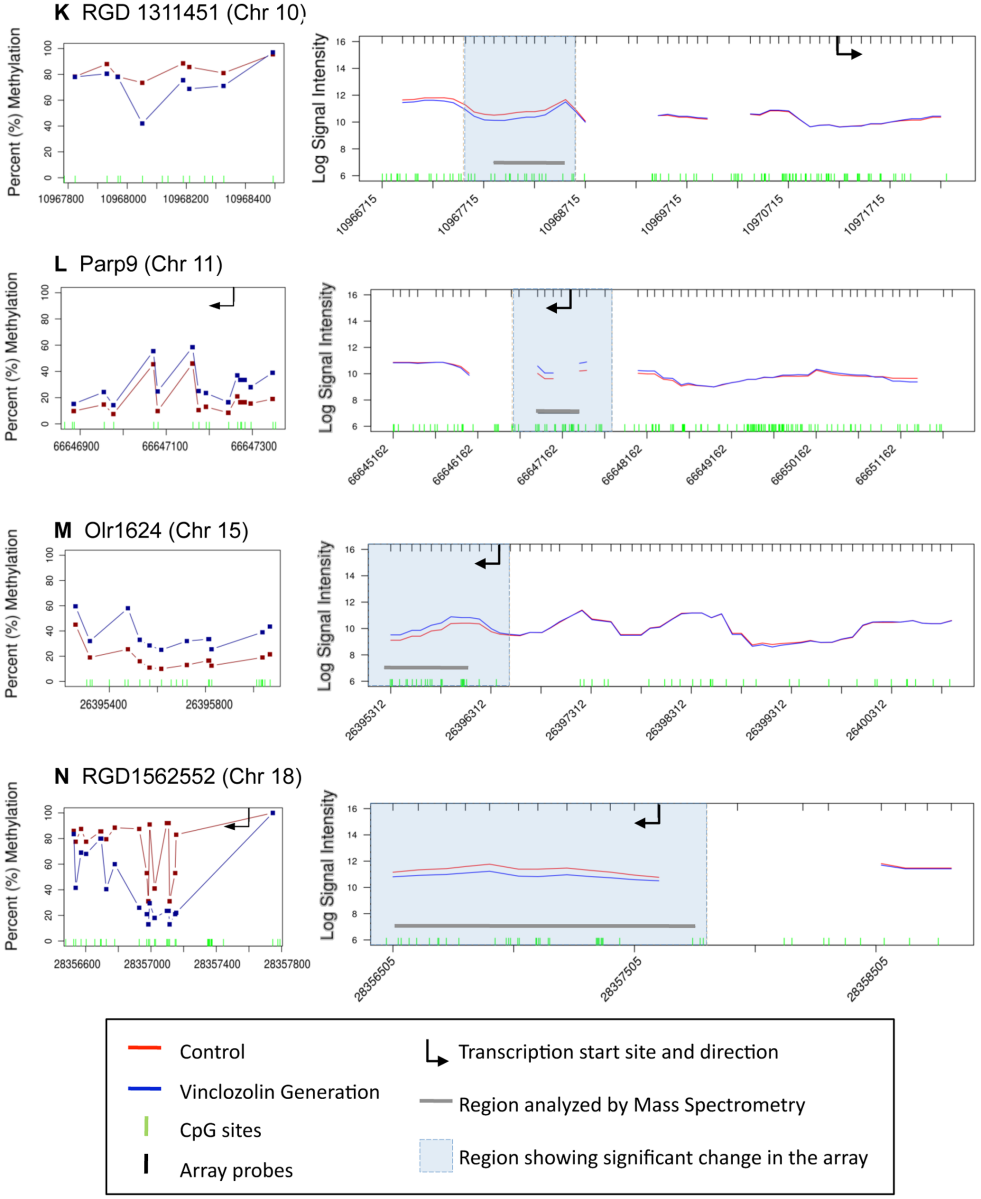
Refer to **Figure 2** Legend.

**Figure 5:**
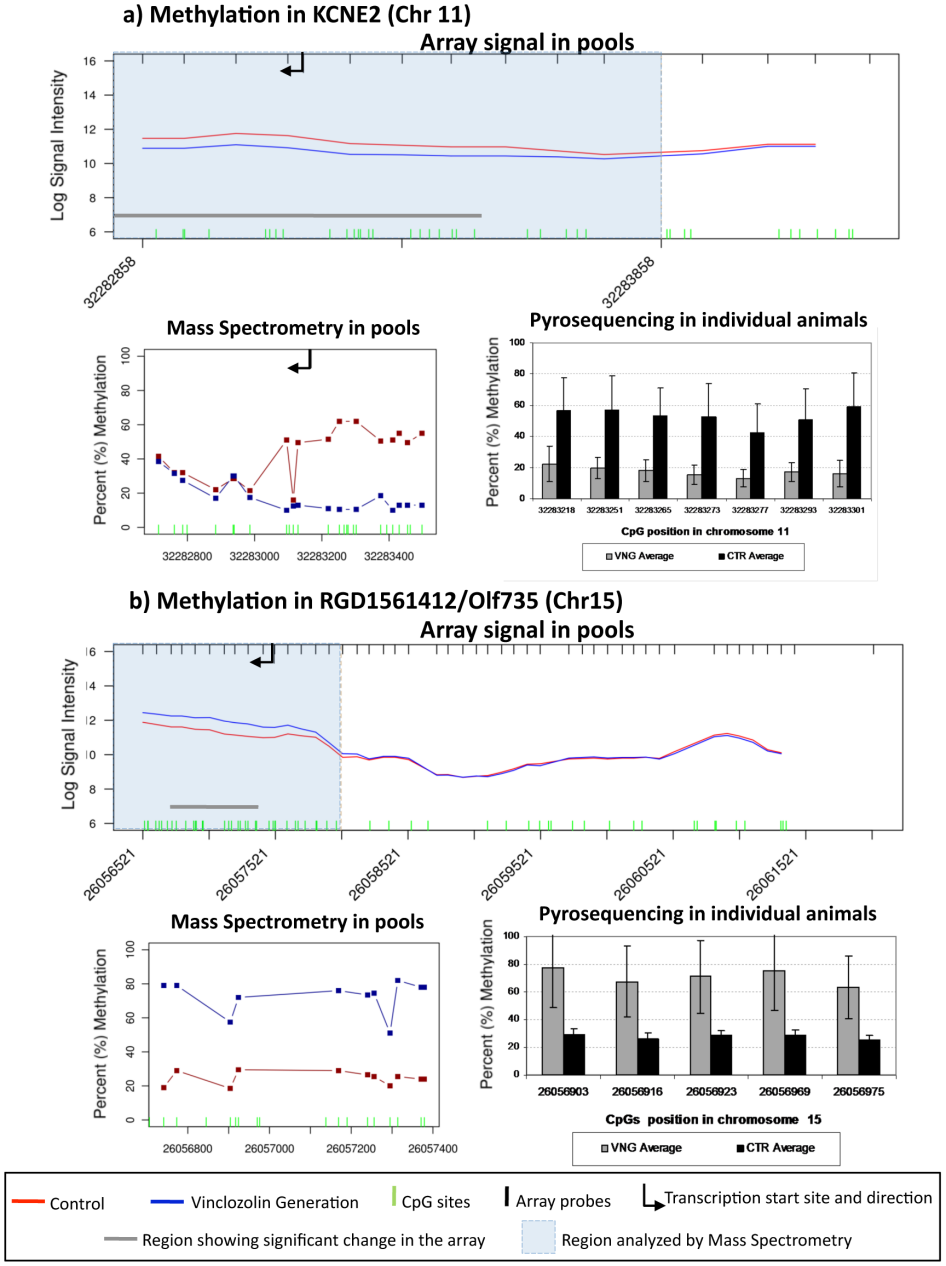
Comparison of the transgenerational methylation change observed between vinclozolin and control in (a) KCNE2 and (b) RGD1561412/Olr735. Analysis of methylation through MeDIP followed by comparative hybridization is graph on top with genome location and log signal intensity presented with shaded area being the differential methylation region. Heavy red line indicates control and blue line indicates vinclozolin and arrow indicates transcriptional start site and direction. The graph on bottom left is the bisulfite mass spectrometry analysis of CpG sites within bar in shaded area of the top graph indicated with percent methylation data presented. Horizontal axis shows chromosomal localizations. In addition, comparison between methylation for these genes in individual animal samples of DNA is shown using pyrosequencing, bottom right graph with percent methylation presented for individual animals (n = 6) mean± SEM for specific CpG in the differential methylation region. Horizontal axis shows chromosomal localizations.

The vinclozolin induced F3 generation epigenetic alterations in DNA methylation of promoter regions in sperm was evaluated in the present study. The strategy used for analyzing genome-wide promoter changes in DNA methylation was immuno-precipitation of methylated fragments with methyl-cytosine antibody followed by promoter tiling microarray chip hybridizations (MeDIP-Chip). This analysis was performed in two pooled DNA samples from two different experiments with F3 generation sperm obtained from control generation animals and compared to two samples obtained from vinclozolin generation animals. Comparison of F3 control versus vinclozolin samples with a comparative hybridization bioinformatics approach produced a list of 52 promoter regions that had statistically significant altered methylation patterns in the sperm (Fig. 1). These 52 differential methylation regions (Supplementary Table S1) were present in 48 different promoters. The gene promoter information, chromosomal location and statistical p-value are listed in Supplementary Table S1. Subsequent quantitative analysis of these regions methylation status was performed using bisulfite treatment followed by mass spectrometry. This method detects changes in methylation in selected regions at an individual CpG resolution [42]. In 16 of these regions changes in methylation patterns were confirmed and measured (Table 1, Fig. 2, 3, 4, 5). In 21 of the regions change in methylation could not be detected in small selected sites (Supplementary Fig. S1), due to the inability to interrogate the entire region because bisulfite primers could not be designed. Therefore, the change could have occurred in adjacent CpG sites not able to be assayed. In the remaining 15 differential methylation regions, no bisulfite primers could be designed for the region so they were not investigated with the bisulfite mass spectrometry procedure. Therefore, 16/48 of the different methylation promoters were confirmed, while the others could not be investigated due to bisulfite analysis limitations. The main limitation in the primer design for bisulfate treated DNA is that primers have to account for the large number of C to T conversions, which create long strings of T that are difficult to design specific primers for every region of interest. Neither the software programs available nor manual analysis for bisulfite primers was successful. A complete discussion on the constraints for design for bisulfite treated DNA has previously been discussed [43]. The list of the 16 genes containing these confirmed promoters, the p-values (paired students t-test, p<0.05), and the direction and the magnitude of the changes are shown in Table 1. The information on all the other 32 promoters is presented in Supplementary Table S1. The changes in DNA methylation observed in these confirmed regions were remarkably concordant between the MeDIP-Chip and the Mass Spectrometry analyses. The genomic locations of these transgenerational changes in DNA methylation (analyzed by these two methods) are shown in Figure 1. The magnitude of the differential methylation with the tiling array and mass spectrometry for the confirmed 16 regions is shown in Figures 2, 3, 4, 5, while the non-confirmed sites are presented in Supplementary Figure S1. Combined data for the two separate experiments of vinclozolin exposure versus controls are depicted in these figures.

**Table 1:** List of 16 regions with confirmed transgenerational changes in methylation between F3 generation sperm samples from Vinclozolin and Control groups.

Genes' Promoter Regions	Chromosome	Description	% Change	Significance (p value)	Methylation Change in F3 VNG Animals
LOC689162	10	Predicted gene	19.4%	3.1E-18	Increased
GPR33	6	G protein-coupled receptors, control chemotaxis	16.6%	9.4E-13	Increased
Btbd16	1	Provisional status	31.7%	1.3E-09	Increased
RGD1561412/Olr 735	15	G protein-coupled receptors, olfactory receptor	47.6%	2.7E-07	Increased
Parp9	11	Developmentally and differentially regulated in several tissues	12.8%	2.7E-08	Increased
Olr1624	15	G protein-coupled receptors, olfactory receptor	18.1%	8.9E-08	Increased
RGD1562552	18	Predicted gene	30.3%	3.3E-05	Decreased
KCNE2	11	Potassium channel, arrhythmia, regulated by estrogen	25.9%	3.3E-05	Decreased
LOC685117	4	Hypothetical protein	34.5%	2.7E-03	Increased
LOC685544	1	Predicted gene	25.8%	5.4E-03	Decreased
Anxa1	1	Inflammatory response, cell proliferation, cancer	13.6%	1.0E-02	Increased
Prr13	7	Provisional status	9.2%	1.4E-02	Decreased
KCNG1	3	Potassium channel, regulated by BMP-2 in smooth muscle	6.8%	1.8E-02	Increased
Eef1d	7	Associated with Copy Number Variation in myotrophic lateral sclerosis	0.7%	1.9E-02	Increased
RGD1560076/RGD1562802	2	Predicted gene	7.1%	3.4E-02	Increased
RGD1311451/Nmral1	10	Belongs to the family of transcription repressors Nmra-like	9.8%	4.2E-02	Decreased

In order to further compare the methylation status of the pooled DNA (in which the MeDIP-Chip was performed) with the DNA from the individual animal rat sperm samples from which these pools were formed, we measured DNA methylation with pyrosequencing [44] for two of the genes presenting change in methylation. The regions chosen were from the predicted gene RGD1561412/Olf735, which had the largest increase in methylation observed among all the annotated confirmed genes (47.6%), and KCNE2, which presented the largest decrease in methylation among the annotated confirmed genes (25.9%). The pyrosequencing analysis confirmed the tiling array and mass spectrometry bisulfite procedures, and replicated the observed alteration in DNA methylation performed in the individual animals compared to the pooled samples for KCNE2 and RGD1561412/Olf735 promoters (Fig. 5). Therefore, the individual animal sperm DNA sample analysis confirmed the differential methylation observed in the pools. DNA methylation levels in the individual animal was statistically significant and consistent with the levels measured in the pooled DNA (Fig. 5), suggesting pooling the sperm DNA did not create artificial differences in DNA methylation compared with the individuals that originated the pools.

### Analysis of the Differential Methylation Genomic Features

Genomic features of the promoter differential DNA methylation regions were analyzed to determine if common sequence features could be identified. Common genomic features can be distinguished by analysis of nucleic acid sequence patterns or motifs. Some of these motifs are known to serve as binding sites for transcription factors [45]. It has been previously shown that motifs derived from methylation-prone sequences are generally associated with CpG islands and are non-randomly distributed along the genome [46]. There are two ways of finding shared motifs in sets of sequences: (i) applying *ab initio* motif discovery algorithms which search for recurring patterns in a set of DNA sequences, or (ii) assessing whether previously characterized motifs present in transcription factor binding site databases are statistically over-represented in the sequences [47]. The *ab initio* tool used in this study was the GLAM2 algorithm (Gapped Local Alignment of Motifs, available online in the MEME suite), which aims to find motifs while considering insertions or deletions, a variable that is not incorporated by other algorithms [48]. GLAM2SCAN was used to search matches of the GLAM2 built motif in specified sequence databases.

**Figure 6:**
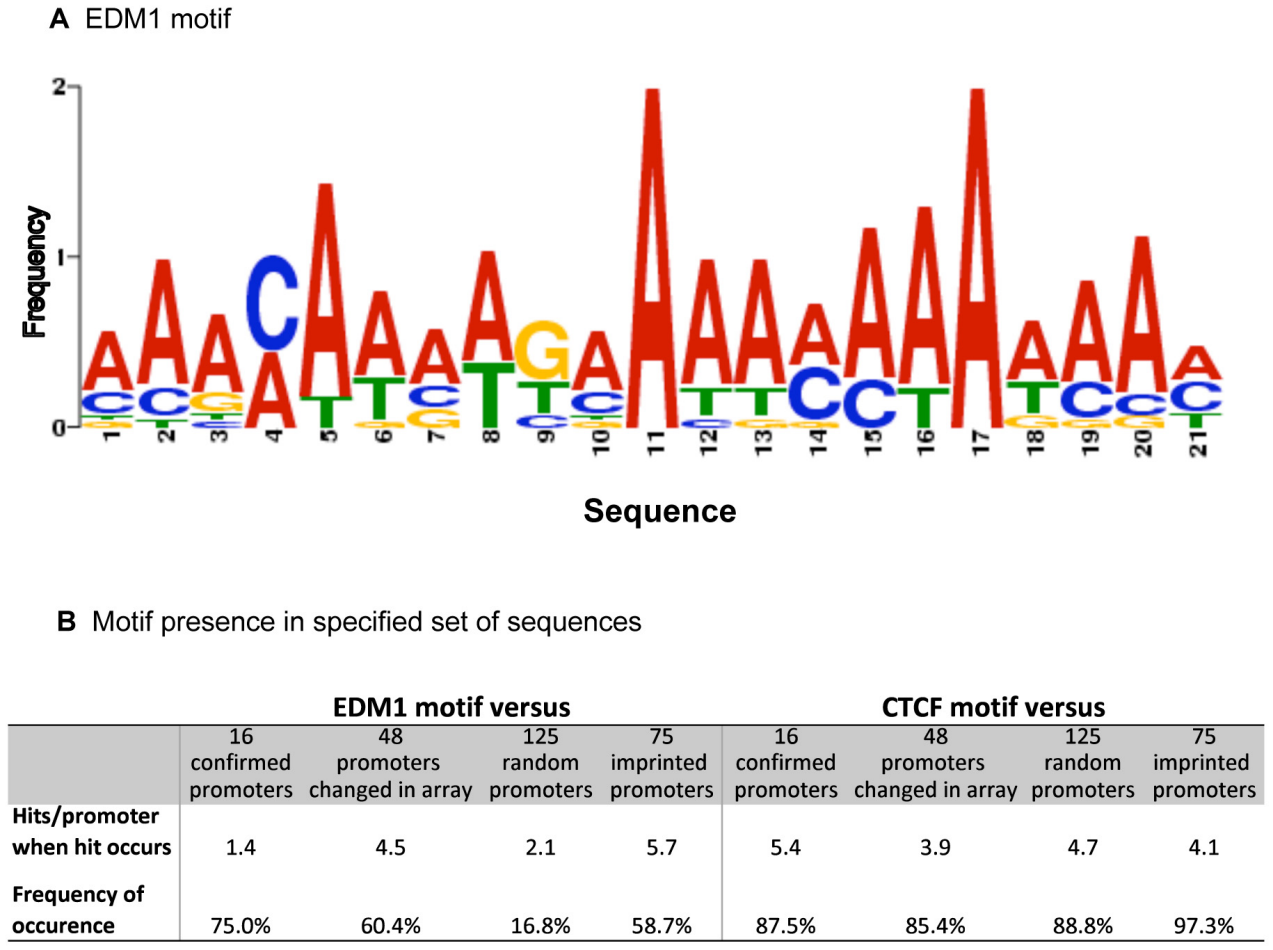
Identification of a DNA sequence motif EDM1 (Environmental Induced Differential Methylation Consensus Sequence 1). (**a**) Logo representation of the motif EDM1. This motif was obtained with the GLAM2 tool of MEME suite from the set of 16 regions that had been confirmed with mass spectrometry to present transgenerational changes in methylation. Glam2 score value for this motif is 194.184 (**b**) Results obtained when scanning EDM1 with GLAM2SCAN for prevalence of matches against four sets of sequences: (i) the 16 promoters containing the regions positively confirmed to be changed, (ii) the 48 promoters containing the regions confirmed to show change in the array, (iii) a set of 125 random promoters, and (iv) a set of 75 imprinted promoter regions from mouse and rat databases. Results shown are from matches in GLAM2SCAN scoring equal or higher than the cut-off value of 20.

The set of 16 differential methylation regions that had been confirmed with mass spectrometry was used as input in GLAM2 and the best motif obtained was used in further comparisons. The logo (e.g. representation of the motif characteristic sequence) obtained for this motif is shown in Figure 6a and the probability matrix provided in Supplementary Table S3. This motif identified with GLAM2 was named EDM1 (Environmental Induced Differential Methylation Consensus Sequence 1). Interestingly, EDM1 contains few possibilities of formation of CpG dinucleotides depending on the sequence, from 0 to 3 CpG. Using GLAM2SCAN, EDM1 was tested for prevalence of matches against four sets of sequences: (i) the 16 promoters containing the regions positively confirmed to be changed, (ii) the 48 promoters containing the 52 regions showing change in the array, (iii) a set of 125 random promoters, and (iv) a set of 75 imprinted promoter regions from mouse and rat databases. With a GLAM2SCAN cut–off score value >20, EDM1 was present in 75% of the positively confirmed set of promoters and in 60.4% of the set of 48 promoters showing change in the tiling array. In contrast, the presence of EDM1 is significantly (p = 0.0001) reduced to 16.8% in the set of 125 random promoters tested (Fig. 6b). In addition, the presence of EDM1 was tested in a set of 75 promoter regions of known mouse and rat imprinted genes (Supplemental Table S4). Interestingly, this analysis showed that EDM1 was present in 58.7% of this set of imprinted gene promoter regions, which is an incidence significantly (p = 0.001) higher than in the set of random promoters, but reduced in comparison with the confirmed promoters, (Fig. 6b and Supplemental Table S4). When EDM1 was present in the promoter of the imprinted genes set, it was at an average of 5.7 hits/promoter, which is a higher frequency than the hits/promoter in the set of confirmed or random promoters (Fig. 6b). Therefore a consensus sequence motif EDM1 was identified and associated with the transgenerational differential methylation.

**Figure 7:**
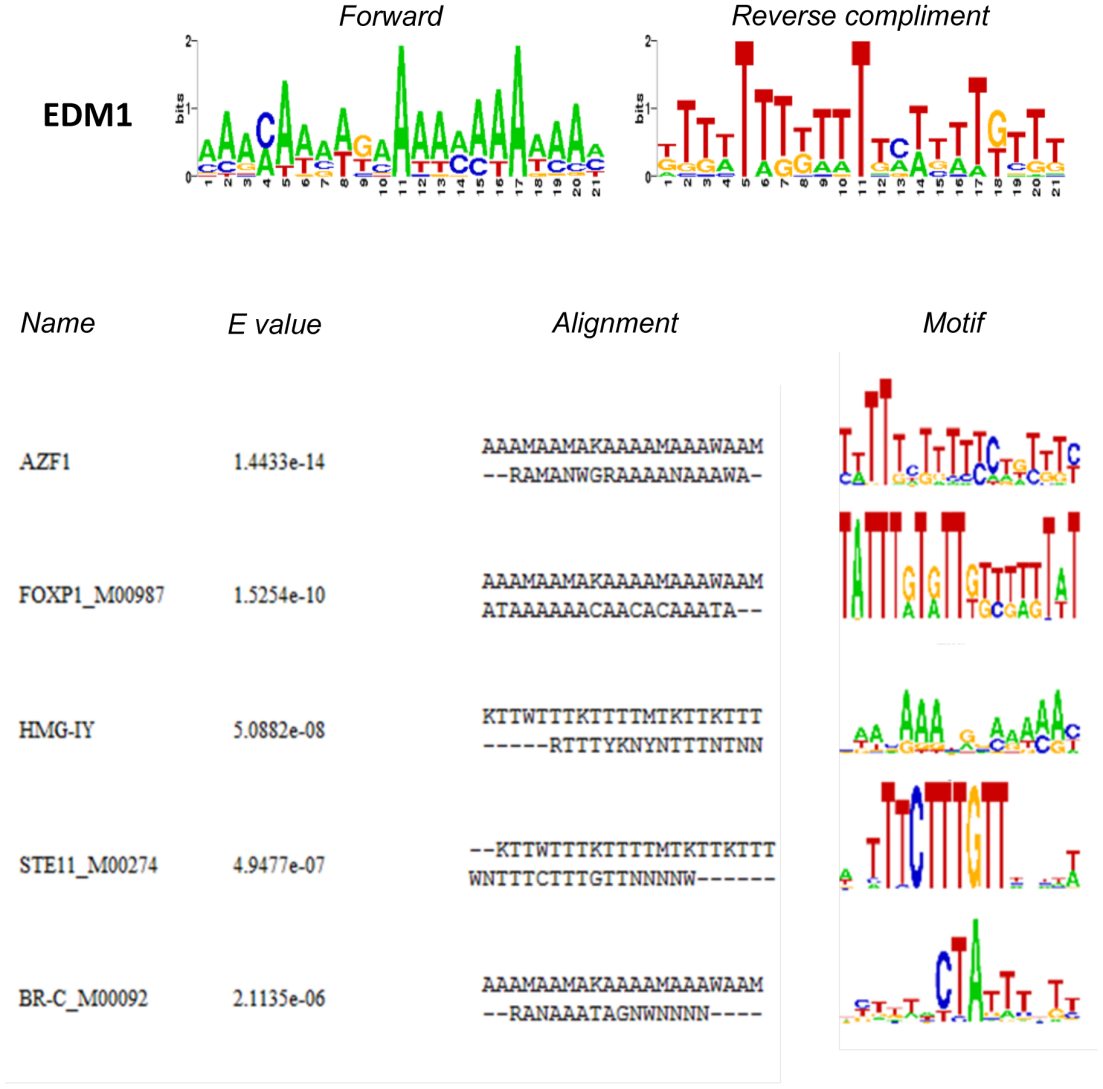
Analysis of similarities between EDM1 and eukaryotic transcription factor binding sites with the STAMP tool. The five top changes and respective significance values are shown with E value, sequence alignment and logo for each.

CTCF binding plays an important role in the establishment of methylation in imprinted genes [49]. Therefore, the possibility that the regions with transgenerational epigenetic changes had an altered prevalence of CTCF binding sites was examined. A previously published consensus motif of CTCF binding sites [50] was tested with the online MEME tool “Find Individual Motif Occurrence” (FIMO) in these sequences. FIMO is an algorithm that aligns a motif (or a set of motifs) to sequences in a database. The occurrence of the CTCF motif was compared among the promoter sets and not found to be different (Fig. 6b). The cut-off value used in FIMO was p<10^−4^. The EDM1 sequence was also compared to the CTCF motif and to other known motifs within the JASPAR database [51] using the STAMP web tool [52]. No significant similarity with the CTCF motif was found (E-value of 0.997) (Fig. 6b). When testing EDM1 for similarities with eukaryotic transcription factor binding sites, it was found that the highest scores of similarity were obtained with the transcription factor binding sites for AZF1 (a zinc-finger factor found in *Saccharomyces cerevisiaes*), FOXP1 (M00987), HMG-IY, STE11 (M00274) and BR-C (M00092) (Figure 7).

Another genomic feature analyzed was repeat elements. An important number of methylated DNA regions are associated with repeat elements [53] and known to have altered DNA methylation patterns in cancer cells [54]. Germ cells have been shown to have repeat elements that are enriched in the DNA methylation associated SNPs [C/T]G or C[G/A] [55]. The promoters with transgenerational differential methylation were analyzed in the online algorithm Repeat Masker in order to interrogate changes in the representation of particular repetitive elements. An increased representation of Long Terminal Repeat (LTR) elements was observed in the confirmed promoter regions (13.01%) as compared to the set of random promoters (6.37%) (Table 2). In addition, variations were found in the representation of the different classes of LTR elements. The most striking difference among these LTR classes was a 10-fold increase in the number of the endogenous retrovirus-like (ERV) class I elements in the confirmed promoters (6%) compared to the random set of promoters (0.64%). An important and statistically significant increase in ERV class II elements (also known as IAPs) was also observed (from 2.34% to 4.49%). Interestingly, in the set of 48 promoters changed in the array, which include confirmed and non-confirmed changes in methylation, the statistically significant difference observed was an increase of ERV class I elements regarding the random set (from 0.64% to 2.47%) (Table 2). Therefore, ERV elements (class I and II) are over represented in the set of promoters that have transgenerational changes in DNA methylation. None of the other features examined were different between the confirmed promoter set and the random promoter set.

**Table 2:** Repeat Masker analysis and comparison between the set of 16 promoter regions confirmed to have transgenerational change in methylation, a set of random 125 promoters and the set of 48 promoter regions showing transgenerational methylation change in the array. Asterisks (*) show significant change regarding the set of random promoters with Fisher exact test (p<0.05).

	16 Promoter Regions Positively Confirmed	Random Background Sample of Promoter Regions	48 Promoter Regions Changed in the Array
**Number of sequences:**	16	1541	48
**Total length:**	110132 bp	9347759 bp	298409 bp
**GC level:**	45.28%	45.83%	44.41%
**Bases masked:**	38027 bp (34.53%)	2675013 bp (28.62%)	92875 bp (31.12%)

	Number of element*	Length (bp)	Percent ofsequence (%)	Number of element*	Length (bp)	Percent of sequence (%)	Number of element*	Length (bp)	Percent of sequence (%)
**SINEs:**	107	13889	12.61	7139	895350	9.58	219	29458	9.87
**Alu/B1**	36	3935	3.57	2468	269240	2.88	69	7559	2.53
**B2-B4**	58	8880	8.06	3344	505688	5.41	115	18526	6.21
**IDs**	12	962	0.87	1013	87450	0.94	27	2284	0.77
**MIRs**	1	112	0.1	313	32857	0.35	8	1089	0.36
**LINEs:**	12	5674	5.15	1582	768699	8.22	61	29494	9.88
**LINE1**	11	5573	5.06	1431	749698	8.02	59	29230	9.8
**LINE2**	1	101	0.09	135	17727	0.19	2	264	0.09
**L3/CR1**	0	0	0	10	986	0.01	0	0	0
**LTR elements:**	29	14326	13.01*	1671	595669	6.37	57	22255	7.46
**ERVL**	4	1169	1.06	253	74435	0.8	6	1535	0.51
**ERVL-MaLRs**	6	1599	1.45	802	241450	2.58	23	6219	2.08
**ERV_classI**	6	6608	6*	155	60213	0.64	8	7360	2.47*
**ERV_classII (IAPs)**	13	4950	4.49*	457	218947	2.34	20	7141	2.39
**DNA elements:**	2	839	0.76	326	58302	0.62	9	2099	0.7
**hAT-Charlie**	2	839	0.76	222	37684	0.4	7	1596	0.53
**TcMar-Tigger**	0	0	0	28	4668	0.05	2	503	0.17
**Unclassified:**	1	108	0.1	56	30219	0.32	2	374	0.13
**Total interspersed repeats:**		34836	31.63		2348239	25.12		83680	28.04
**Small RNAs:**	3	260	0.24	322	32732	0.35	6	591	0.2
**Satellites:**	0	0	0	70	11909	0.13	0	0	0
**Simple repeats:**	42	2561	2.33	3494	210711	2.25	105	7134	2.39
**Low complexity**	13	370	0.34	1568	71569	0.77	39	1470	0.49

### Copy Number Variation Analysis

**Figure 8:**
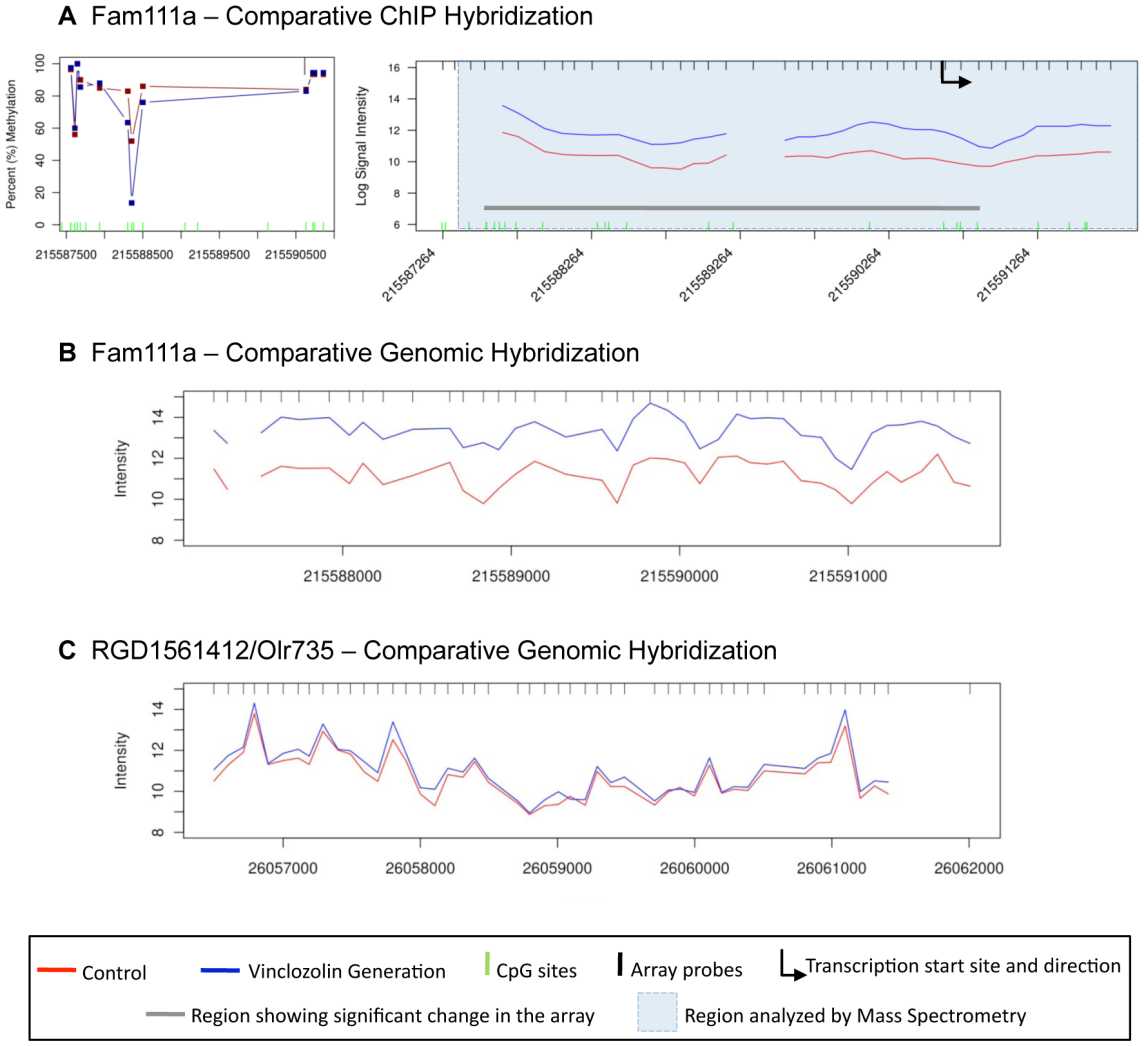
Identification of differential DNA methylation site and associated copy number variation. (**a**) Comparison of the transgenerational methylation change observed between vinclozolin F3 generation sperm (VNG) and control F3 generation sperm (CTR) in Fam111a. Analysis of methylation through MeDIP followed by comparative hybridizations of methylated DNA immuno-precipitations and mass spectrometry analyses are shown. (**b**) Comparative genomic hybridization signals between F3 generation VNG and CTR sperm are shown for Fam111a. (**c**) Comparative genomic hybridization signals between VNG and CTR are shown for RGD1561412/Olr735.

The consistency of the DNA methylation measurements observed between the MeDIP-Chip and bisulfite mass spectrometry analyses was monitored to validate procedures. In the case of the candidate promoter Fam111a the analysis did not compare. This region presented a highly significant change in methylation in the MeDIP-Chip promoter array between the F3 generation control and vinclozolin generation sperm (Supplementary Table S1), however, its methylation level was unchanged in the mass spectrometry methylation analysis, which had approximately 90% levels of DNA methylation (Fig. 8a). The hypothesis was investigated that this inconsistent result could be attributed to a gene copy number variation (CNV). Copy number variation can be associated with particular genomic regions and the incidence of disease [56], and can vary among different populations of humans [57]. The potential presence of a CNV in the promoter region of Fam111a was determined by comparing the signal of the tiling array with genomic DNA inputs between vinclozolin and control samples (i.e. comparative genomic hybridization), CGH. Interestingly, it was found that the signal in the array hybridization versus input was significantly higher in vinclozolin than in control F3 generation sperm (Fig. 8b). This indicates an increase in copy number in the vinclozolin group for the Fam111a promoter. This observation was not found in any other gene promoters in which the difference between vinlcozolin and control was highly significant, such as the example case for RGD1561412/Olr735 (Fig. 8c). No other promoter region in the genome was found to contain a CNV (data not shown), but genome wide analysis for regions outside promoters is now required to assess the specificity of the CNV identified.

## Discussion

An environmental exposure that can influence a critical developmental period (e.g. embryonic development) for an organ system can later in life promote an adult onset disease [7], [9], [58], in part due to alterations in the epigenome. In the event a permanent alteration in the germ line epigenome develops, a transgenerational phenotype is possible [1], [14]. Previous reports demonstrated that the environmental endocrine disruptor vinclozolin can promote a transgenerational disease phenotype and altered DNA methylation in the germ line [9]. Vinclozolin is a commonly used fungicide in the fruit (e.g. wine) industry [58] and its two major metabolites (M1 and M2) are anti-androgenic compounds [59]. These initial findings on the transgenerational epigenetic effects of vinclozolin are expanded in the present study by interrogating genome-wide methylation changes in the promoter regions of F3 generation sperm. Observations identify a number of genes that have their promoter DNA methylation patterns altered in the F3 generation male germ line, following an early embryonic (in utero) exposure of the F0 gestating female to the endocrine disruptor vinclozolin. In the genome wide promoter analysis only 48 different promoters were found to have differential DNA methylation in the F3 vinclozolin generation sperm. Some promoters had multiple regions such that 52 total regions were identified. The comparative hybridization MeDIP-Chip tilling array procedure was reproducible and reliably identified the differential methylation with an average region of 500–600 bp in size. The MeDIP-Chip analysis does not map the alteration in DNA methylation at the CpG level, but requires an alternate procedure such as bisulfite conversion of cytosine residues followed by mass spectrometry [42]. A limitation to this bisulfite mass spectrometry procedure is that only limited sites can be interrogated due to the inability to design bisulfite PCR primers for all regions. This is due to the complexity of partially converting a 4 base genome sequence to a 3 base sequence. Analysis presented in Figures 2, 3, 4 and Supplementary Figure S1 demonstrates the sub-regions within each candidate that was mapped and shows the sequence that could not be mapped. From the 52 candidate regions identified 16 were confirmed with bisulfite mass spectrometry, 21 were unconfirmed with the specific site analyzed and 15 were not able to be analyzed. The 21 unconfirmed regions did not show changes in the CpG methylation sites interrogated, but adjacent sequences in the same region could not be interrogated due to limitations of primer design for bisulfite treated DNA. These regions were categorized as unconfirmed. Therefore, the 36 unconfirmed candidates are still viable differential methylation sites, but will require more advanced technology to map the entire region at the CpG level resolution. Neither the computer software programs available nor manual procedures used could generate the bisulfite promoters needed for these 36 promoters. Therefore, further analysis of these 36 unconfirmed regions is needed. The current study focused on the 16 confirmed sites for follow up analysis.

The differential DNA methylation regions identified were found in the F3 generation sperm epigenome following vinclozolin exposure of the F0 generation [1]. As previously described [7], the presence of a phenotype and epigenetic modification in the F3 generation following exposure of a gestating F0 generation female constitutes an epigenetic transgenerational phenotype and inheritance mechanism. Studies are now ongoing to investigate the alterations in the corresponding F1 and F2 generation sperm epigenome for comparison with the F3 sperm epigenome alterations reported in the current study. Since the F1 and F2 generations are not directly defined as transgenerational [7], it will be interesting to assess the similarities of sperm epigenome alterations between those generations. In addition, a consistent epigenetic alteration would be predicted for subsequent generations to the F3 (e.g. F4). All these studies are beyond the scope of the current study, but will be useful to clarify that the differential DNA methylation sites identified in the current study are consistently transmitted transgenerationally through the sperm. The current study documents a transgenerational epigenetic effect from an environmental exposure on the sperm epigenome. This indicates the epigenetic modification was not erased or eliminated during early embryonic development nor during germline programming at gonadal sex determination. This germ-line mediated epigenetic transgenerational inheritance phenomena now needs to be further investigated on a mechanistic level.

The 16 confirmed differential methylation regions were further investigated and discussed below. The DNA methylation patterns of the promoters of a number of genes were found to be transgenerationally transmitted including the annotated genes GPR33, KCNE2, ANXA1, Olr735, Olr1624, Parp-9, KCNG1, Eef1d and Nmral1. GPR33 is an orphan chemo-attractant G protein-coupled receptor that has been identified as an inactivated pseudogene involved in leukocyte chemotaxis in humans, as well as in several great ape and rodent species [60]. Interestingly, in species where this inactivation occurred, no genetic variation in the gene is observed [60]. Epigenetic inactivation of this gene would be a plausible alternate hypothesis to consider in the absence of genetic variation. KCNE2 encodes a single transmembrane domain protein that modulates a variety of K^+^channel functions in several tissues. Alterations in KCNE2 associates with human cardiac arrhythmogenesis and long QT syndrome [61], as well as with down regulation of two major components of murine cardiac action potential repolarization currents and changes in gastric secretion [62]. In addition, KCNE2 expression is estrogen dependent [63] and is down regulated in gastric cancer while its over expression suppresses cell proliferation and tumorgenesis in a gastric cancer cell line [64]. KCNG1 is another gene encoding a potassium channel that is included in the confirmed regions. KCNG1 has been shown to be up-regulated when treating human cells with the Bone Morphogenetic Protein-2 (BMP-2) factor [65].

The promoters of two olfactory receptor genes were found to be transgenerationally altered in regards to their DNA methylation. These were Olr1624 and the recently annotated gene Olr735. This finding correlates with previous data showing that early exposure to vinclozolin affects sexual selection (i.e. mate preference) in rats [10] and also alters the expression of genes related to olfactory transduction in the male amygdale, including Camk2a, Camk2d and Prkg2 [25]. The ANXA1 gene was identified and previously the Annexin V protein has been shown to be increased in the sperm of mice exposed to vinclozolin during gestation [66]. ANXA1 expression in prostate carcinogenesis correlates with enhancing tumor aggressiveness through increasing IL-6 expression and activity [67]. Interestingly, vinclozolin was found to promote transgenerational prostate disease [68].

The confirmed gene Parp-9, in turn, is a macro-domain containing poly (ADP-ribose) polymerases involved in transcriptional regulation in response to immunoregulatory cytokines. Parp-9 has been shown to be developmentally regulated in mouse and to present differential expression in tissues, being expressed in the thymus and specific regions of the brain and gut [69]. RGD1311451/Nmral1 is another of the confirmed genes that would also have a role in regulation of transcription. Interestingly, the RGD1311451/Nmral1 transcript belongs to a family of transcriptional repressors (NmrA-like) that regulate expression through discrimination between oxidized and reduced dinucleotides and are known as ‘redox sensors’ [68].

In order to identify genomic elements associated with the transgenerational differential DNA methylation sites, we investigated if a consensus DNA sequence could be identified among the regions with confirmed transgenerational epigenetic change. A bioinformatics tool (GLAM2) was used to identify a consensus sequence and an Environmental Induced Differential Methylation Consensus Sequence 1 (EDM1) motif was identified. The EDM1 sequence was present in 75% of the positively confirmed promoters, in 60.4% of the 48 promoters with differential methylation, while being present in only 16.8% of a random set of promoters. The prevalence of the EDM1 sequence in future environmentally induced differential DNA methylation sites will need to be confirmed and further investigated. In the event a consensus sequence may be identified for environmental alterations in the epigenome, the sequence may be used to cross species and help select sensitive genomic sites. The identification of EDM1 suggests a consensus sequence may be involved in promoting the regions sensitivity to or maintenance of the epigenetic transgenerational differential DNA methylation. Comparison of EDM1 with known eukaryotic transcription factors revealed similarities with AZF1(a), FOXP1 (M00987), HMG-IY, STE11 (M00274) and BR-C (M00092). AZF1 is a zinc-finger factor found in *Saccharomyces cerevisiaes* known to bind to DNA elements with the sequence AAAAGAAA [70]. In mammals, zinc fingers proteins regulate normal cell proliferation and differentiation through development, acting as tumor suppressors or oncogenes [71]. FOXP1 is thought to have a role in hepatocarcinogenesis that would be mediated by epigenetic mechanisms. FOXP1 is one of the targets of miR-1 micro RNA. Activation of mir-1-1 gene occurs through demethylation, leading to reduced expression of FOXP1, which is up-regulated in hepato-cellular carcinogenesis [72]. HMG-IY is an important intermediate factor in the pathway leading to proliferation in the human pancreatic adenocarcinoma cell line [73]. STE11 is well characterized in *Saccharomyces cerevisiaes*, where it is known to be part of the Hog1 MAP (mitogen-activated protein) kinase pathway, which is activated after exposure to high osmolarity [74]. BR-C (Broad Complex) is important for metamorphosis in drosophila and silkworms, and responds to hormonal signaling pathways [75], [76]. Further investigation on the hormonal and epigenetic roles of these transcription factors is needed in order to elucidate possible pathways in which environmental stimuli produces epigenetic transgenerational changes in the male germ line.

The transgenerational differential methylation consensus sequence EDM1 identified was present at relatively high frequency (60.4%) in the regions found, and at a low (16.8%) frequency in the random set of promoter regions compared. Interestingly, analysis of mouse and rat imprinted gene promoters (Supplemental Table S4) also revealed a high frequency (58.7%) of the presence of EDM1. The possibility that the EDM1 motif may be involved in the transgenerational differential methylation and DNA methylation in imprinted genes will need to be addressed in the future. Mechanistically this may reveal why these sites may be more sensitive to environmental changes in the epigenome compared to the rest of the genome. Interestingly, EDM1 was not similar to a CTCF consensus motif. This comparison was performed due to the importance of CTCF in methylation of imprinted genes [49]. The presence of the CTCF motif in the promoters with differential methylation and the random set of promoters was the same and suggests that CTCF binding is likely not the mechanism involved in the transgenerational changes in methylation. Recent findings also suggest that CTCF binding is not the mechanism for establishment of methylation in the male germ line but only in somatic cells and female germ line [77], [78], [79]. Other features analyzed in the promoter regions that presented transgenerational change in methylation were repeat elements. An increase in ERV elements class I and class II (also known as IAPs) was found in the regions with methylation change. It is important to note that IAPs have been shown to have their methylation patterns affected by nutrition in the Agouti mouse model, leading to a concomitant change in coat color [80], [81]. Recent evidence suggests that methylation in IAPs is more resistant to erasure than other genomic regions [31], [82]. This would lead to the speculation that if an environmental insult is capable of altering methylation in IAPs, in the further generations this methylation would be resistant to be changed, except by exposure to another significant environmental insult.

The ability of an environmental factor to promote an epigenetic transgenerational phenotype has been termed epigenetic transgenerational inheritance [1], [9], [83], [84], [85], [86]. In the event the germ line has a permanent epigenetic alteration, a transgenerational epigenetic phenotype may be transmitted [1]. The possibility that an initial epigenetic event may promote a secondary genetic alteration has been proposed as a potential mechanism for transgenerational inheritance [83], [84]. Therefore, the degree epigenetic versus genetic molecular events are involved needs to be assessed [9], [84]. The current study demonstrates transgenerational epigenetic alterations (i.e. differential methylation) in the promoters for a number of genes. Interestingly, an event of copy number variation (CNV) was also identified in one promoter, Fam111a. The possibility that the epigenetic changes that developed in early F1 generations promoted an alteration in the CNV of Fam111a that developed at the F3 generation suggests secondary epimutations involving genetic alterations is possible [83]. In the event an environmental factor promoted an epigenetic alteration that subsequently promoted a genetic change, such as a SNP polymorphism or CNV, a combination of epigenetic and genetic processes will be involved in the transgenerational inheritance. The current observations support this hypothesis and further genome wide approaches are now needed to elucidate the molecular mechanisms involved.

## Materials and Methods

### *In vivo* procedures

Gestating outbred Harlan Sprague–Dawley mother rats from timed pregnant colonies housed at the Washington State University vivarium were given intra-peritoneal injections of vinclozolin (100 mg/kg/day) from embryonic day 8 to 14 of gestation (i.e., F0 generation) as previously described [1], [14]. The sperm-positive vaginal smear date was taken as embryonic day 0. Gestating control mothers (i.e., F0 generation) received vehicle alone (i.e., sesame oil and DMSO). At least eight lines (individual F0 injected females) were generated for controls and eight lines for vinclozolin generations for these analyses. The F1–F3 generation animals derived from a vinclozolin-exposed F0 mother are referred to as vinclozolin-generation animals, while those from control F0 mothers are identified as control generation animals. Adult F1 vinclozolin-generation (offspring from F0 mothers) males were bred to F1 vinclozolin-generation females to generate the F2 vinclozolin generation. Adult F2 vinclozolin-generation males were bred to F2 vinclozolin-generation females to generate the F3 vinclozolin-generation. Rats for the control (vehicle treated) groups (i.e., generations F1–F3) were bred in the same manner for all the generations. No inbreeding or sibling crosses were generated. All procedures were approved by the Washington State University Animal Use and Care Committee (IACUC approval # 02568-014).

### Sperm extraction, DNA isolation and methylated DNA immunoprecipitation

Sperm were extracted from the cauda epididymus as previously described [19], from a total of six F3 vinclozolin generation animals and six F3 control generation animals. Each experimental group contained three animals from each control and vinclozolin exposures, which were one year apart. Therefore, animals were divided into four groups: three vinclozolin generation animals and three control-generation animals from the first exposure (V1 and C1 respectively), and three vinclozolin generation animals and three control generation animals from the second exposure (V2 and C2 respectively). Sperm samples were resuspended in 1 ml buffer 0.5M tris-HCl, pH 8, 0.5M EDTA, 10% SDS and treated with 100 ml proteinase K (20 mg/mL) and 100 ml DDT (0.1 M) at 55 C for 5 hours. DNA was isolated by phenol:chloroform:isoamyl alcohol extraction method, washed with 70% ethanol and resuspended. Sperm DNA was pooled for each of the four above mentioned groups, adding equal amounts of sperm DNA from each animal to the pools. The addition of equal amounts of DNA from each individual animal to the pools was confirmed to represent each animal equally. Therefore, a total of four pools of sperm DNA (C1, C2, V1 and V2), each including DNA from 3 animals, were used as samples for further immunoprecipitation (MeDIP) of methylated DNA fragments. This design that includes pooled DNA for IP was chosen in order to include animal variability in the experimental procedures. The MeDIP was performed as follows: 6 ug of genomic DNA was subjected to series of three 20 pulse sonications at 20% amplitude and the fragment size verified through 2% agarose gels with approximately a 500 kb size; the sonicated genomic DNA was resuspended in 350 ul TE and denaturated for 10 min at 95°C and then immediately placed on ice for 5 min; 100 ul of 5X IP buffer (50 mM Na-phosphate pH7, 700 mM NaCl, 0.25% Triton X-100) was added to the sonicated and denatured DNA. An overnight incubation of the DNA was performed with 5 ug of antibody anti-5-methylCytidine monoclonal from Diagenode S.A at 4°C on a rotating platform. Protein A/G beads from Santa Cruz were prewashed on PBS-BSA 0.1% and resuspended in 40 ul 1X IP buffer. Beads were then added to the DNA-antibody complex and incubated 2 h at 4°C on a rotating platform. Beads bound to DNA-antibody complex were washed 3 times with 1 ml 1X IP buffer; washes included incubation for 5 min at 4°C on a rotating platform and then centrifugation at 6000 rpm for 2 min. Beads-DNA-antibody complex were then resuspended in 250 ul digestion buffer (50 mM Tris HCl pH 8, 10 mM EDTA, 0.5% SDS) and 3.5 ul of proteinase-k (20 mg/ml) was added to each sample and then incubated overnight at 55°C on a rotating platform. DNA purification was performed first with phenol and then with chloroform:isoamyl alcohol. Two washes were then performed with 70% ethanol, 1 M NaCl and glycogen. ChIP selected DNA was then resuspended in 30 ul TE buffer. In order to account for ChIP bias that would interfere with the comparative hybridization, and to homogenize intra-sample variability, several parallel IPs were performed for each sample and then 8 successful IPs were pooled per sample; therefore one pool of IP material was made per group of pooled sperm DNA (C1, C2, V1 and V2).

### Tilling Array MeDIP-Chip Analysis

Roche Nimblegen's Rat ChIP 385K Promoter 2 array set (Catalog Number: 05224195001) was used, which contains at total of 777,752 probes (388,901 on array 1 and 388,853 on array 2), with probe sizes ranging from 50-75mer in length and a median probe spacing of 105 bp. The array set tiles 15,911 regions encompassing the promoters of 22,833 primary transcripts (approximately 4,500 bp upstream and 1,125 bp downstream from transcription start site). This represented approximately 97Mb and 3.7% coverage of the rat genome. Two different comparative (ChIP vs ChIP) hybridizations experiments were performed, each encompassing 4 samples (2 treatment and 2 control) and 4 array sets. The first was a dye balance design as follows: C1 in the green channel against V1 in the red channel; V1 in the green channel against C2 in the red channel; C2 in the green channel against V2 in the red channel; V2 in the green channel against C1 in the red channel. The second one was a dye swap design as follows: C1 in the green channel against V1 in the red channel; V1 in the green channel against C1 in the red channel; C2 in the green channel against V2 in the red channel; V2 in the green channel against C2 in the red channel.

### Bioinformatic and statistic analyses of chip data

For each hybridization experiment, raw data from both the Cy3 can Cy5 channels were imported into R (R Development Core Team (2010), R: A language for statistical computing, R Foundation for Statistical Computing, Vienna, Austria. ISBN 3-900051-07-0, URL http://www.R-project.org), checked for quality and converted to MA values (M = Cy5-Cy3; A =  (Cy5+Cy3)/2). Each array exhibited quality issue where the Cy3 channel failed to decline, relative to the Cy5 channel, in a linear manner with intensity and GC. In order to combat this quality issue the following normalization procedure was conducted. Within each array, probes were separated into groups by GC content and each group was separately normalized using the loess normalization procedure. This allowed for groups with optimal GC content, which exhibited a reduced quality issue, to receive a normalization curve specific to that group. After each array was normalized within array, the arrays were then normalized across arrays using the A-quantile normalization procedure.

Following normalization each probe within each array was subjected to a smoothing procedure, whereby the probe's M values were replaced with the median value of all probe M values within a 600 bp window is at least 4 probes were present in the window and NA if less than 4 probes were present in the window. Each probe's A values were likewise smoothed using the same procedure. Following normalization and smoothing each probe's M value represents the median intensity difference between Cy5 and Cy3 of a 600 bp window. Significance was assigned to probe differences between vinclozolin generation and control generation by calculated by the median value of the intensity differences as compared to a normal distribution scaled to the experimental mean and standard deviation of M. Regions of interest were then determined by combining consecutive probes with significance p-values less than 1e-7. Significance was assigned to probe differences between vinclozolin generation and control generation by calculating the median value of the interesting differences as compared to a normal distribution scaled to the experimental mean and standard deviation of the mean. A Z-score and P-value were computed from that distribution (i.e. the tails) with the use of R code analysis. The statistically significant differential DNA methylations were identified and P-value associated with each region presented. Each region of interest was then annotated for gene and CpG content.

Regions chosen for validation were then described as overlapping regions of interest between the 2 hybridization experiments. This list was further reduced to those overlapping regions with an average intensity value exceeding 9.5 (log scale), at least one 100 bp region with two CpGs or a CpG density>1.

Copy Number Variation was determined by comparing directly the normalized genomic DNA of the same four pooled samples on the same microarray platform, from a separate ChIP vs. Input experiment (not discussed here). A consistent difference in normalized intensity values across the entire promoter region was determined to be evidence for a CNV.

### Individual CpG Methylation Analyses

Each pool of sperm DNA (same as those used for the ChIPs) or individual animal sperm DNA was bisulfite treated according to the method described by Clark et al. [43]. Candidates were chosen based on the promoter tilling array data and individual CpG methylation was measured in the selected regions. In the pools, mass spectrometry analysis was performed for the detection and quantitative analysis of DNA methylation [42]. This system uses homogeneous base specific cleavage (MassCLEAVE) and matrix-assisted laser desorption/ionization time-of-flight mass spectrometry [42]. Mass spectrometry analyses of methylation was performed by Sequenom Inc. protocols by the University of Arizona, Genetics Core Laboratory, Tucson Arizona. Amplicons analyzed are described in the Supplementary Table S2. In the individual animal sperm that generated the pools, pyrosequencing analysis was also performed. Pyrosequencing is a sequencing-by-synthesis method that quantitatively monitors the real-time incorporation of nucleotides through the enzymatic conversion of released pyrophosphate into a proportional light signal [44]. Quantitative determination of CpG DNA methylation in these regions requires using PCR products from previously bisulfite treated DNA. For RGD1561412/Olr 735 primers used were: forward, 5′ TGTTTAGTTTATTGGGGTTATAGAA 3′; reverse, 5′ ACCTCAAAATCATAAATAACCACC 3′, biotinilated at the 5′ end; sequencing reverse: 5′ TTGGGGTTATAGAAAGGTA 3′. For KCNE2 primers used were: forward, GTAATTTAGTTTTTAAGGAGGTGTTGA 3′; reverse, 5′ TTCCTCACAAAAATACTAATATCCC 3′, tailed at the 5′ end; sequencing forward, 5′ TTTAGGAGGTGTTCATTATA 3′. PCR reactions were performed in a final volume of 30 µl, containing 3 µl 10× PCR Qiagen Hot Star buffer, dnase free water adjusted to 30 µl final volume, 0.3 µl of the forward primer (10 µM) plus 0.3 µl of the reverse primer for KCNE2 (10 µM) or 0.12 µl of the reverse tailed primer (2.5 µM) and 0.27 µl of universal biotinilated (10 µM) primer for RGD1561412/Olr 735, 1.2 µl of dNTPs (10 mM), 0.4 µl (0.5 mg/ml) of ET SSB (Biohelix), 0.15 µl Qiagen Hot Star taq polymerase, 1 µl of bisulfite treated DNA template (20 ng/µl). PCR was programmed as follows: 95°C/15 min×1 cycle; 95°C/30 sec, annealing temperature/30 sec, 72°C/30 sec, ×45 cycles; 72°C/5 min×1 cycle. Annealing temperatures was 60°C RGD1561412/Olr 735 and 63°C KCNE2. PCR products were then sent to EpigenDx (Worchester MA) to perform the pyrosequencing analyses. Paired t-test (2-tails) was performed with the Biostat 9.0 software (Analystsoft, Inc.) in pyrosequencing and mass spectrometry analyses, p<0.05 was considered as a significant difference.

### Analysis of sequence features

For *ab initio* discovery of motifs in the regions (inside promoters) that presented a transgenerational change in DNA methylation, GLAM2 algorithm, available at MEME suite [87], were used. GLAM2SCAN, another tool in MEME suite, was used to search for matches of the GLAM2 built motif in our set of sequences. STAMP [52] was used to detect similarities among motifs we have obtained and also to test for similarities of these motifs with a database of transcription factors (eukaryotic). The cut-off p-value used was 10^-4^. Statistical comparison of the incidence of the motif found with GLAM2 among the different sets of promoters was performed with Fisher exact test. RepeatMasker (A.F.A. Smit, R. Hubbey and P. Green, RepeatMasker at http://repeatmasker.org) was used to detect for differences in the presence of repeat elements among our set of sequences.

## Supporting Information

Figure S1 Comparison of the methylation signal in regions where transgenerational methylation change could not be confirmed between vinclozolin and control. Analysis of methylation through MeDIP followed by comparative hybridization (right graph) and through bisulfite mass spectrometry (left graph) is shown for each gene (a-r). Horizontal axis shows chromosomal localizations. For the (c) and (h) genes the probe density for hybridization signal was insufficient to allow a tiling graph to be generated in the shaded regions.
(2.90 MB PDF)

Table S1 List of 52 regions (belonging to 48 promoters) showing transgenerational methylation change in the array and their characteristics. Confirmation status, gene name (Rat Genome Database, RGD), known description of function, RGD and Entrez identification numbers, raw p values and chromosome localizations are listed.
(0.09 MB PDF)

Table S2 List of amplicons used for mass spectrometry methylation analysis, with information on length, number of CpG sites analyzed, location and strand amplified.
(0.96 MB PDF)

Table S3 Letter probability matrix for EDM1. Numbers indicate the probability of occurrence of the nucleotide for each position in the motif.
(0.02 MB PDF)

Table S4 List of promoters of known imprinted genes that were tested against EDM1.The table includes location of the gene, information regarding if the promoter was from Mus musculus or Rattus norvegicus and also information regarding if EDM1 presented at least one hit inside the promoter region of that gene. The list was compiled from the ‘Catalogue of Parent of Origin Effects: Imprinted Genes and Related Effects’ from the University of Otago, New Zealand. The 75 promoter used were selected because there was information on their Transcription Start Site at the NCBI Nucleotide tool and on the direction of their transcription at the NCBI Gene tool. The promoter region of each gene was calculated by adding 5000 bp upstream and 1200 downstream of the Transcription Start Site (ATG) obtained from the NCBI Nucleotide tool.
(0.08 MB PDF)
