# Supplementary material for: Epigenetic Transgenerational Actions of Vinclozolin on Promoter Regions of the Sperm Epigenome
Source: PLoS One. 2010 Sep 30;5(9):e13100. doi: 10.1371/journal.pone.0013100 (PMC2948035; doi:10.1371/journal.pone.0013100)
Supplement: Figure S1 — Comparison of the methylation signal in regions where transgenerational methylation change could not be confirmed between vinclozolin and control. Analysis of methylation through MeDIP followed by comparative hybridization (right graph) and through bisulfite mass spectrometry (left graph) is shown for each gene (a-r). Horizontal axis shows chromosomal localizations. For the (c) and (h) genes the probe density for hybridization signal was insufficient to allow a tiling graph to be generated in the shaded regions. (2.90 MB PDF) [file pone.0013100.s001.pdf]

Supplementary Figure S1

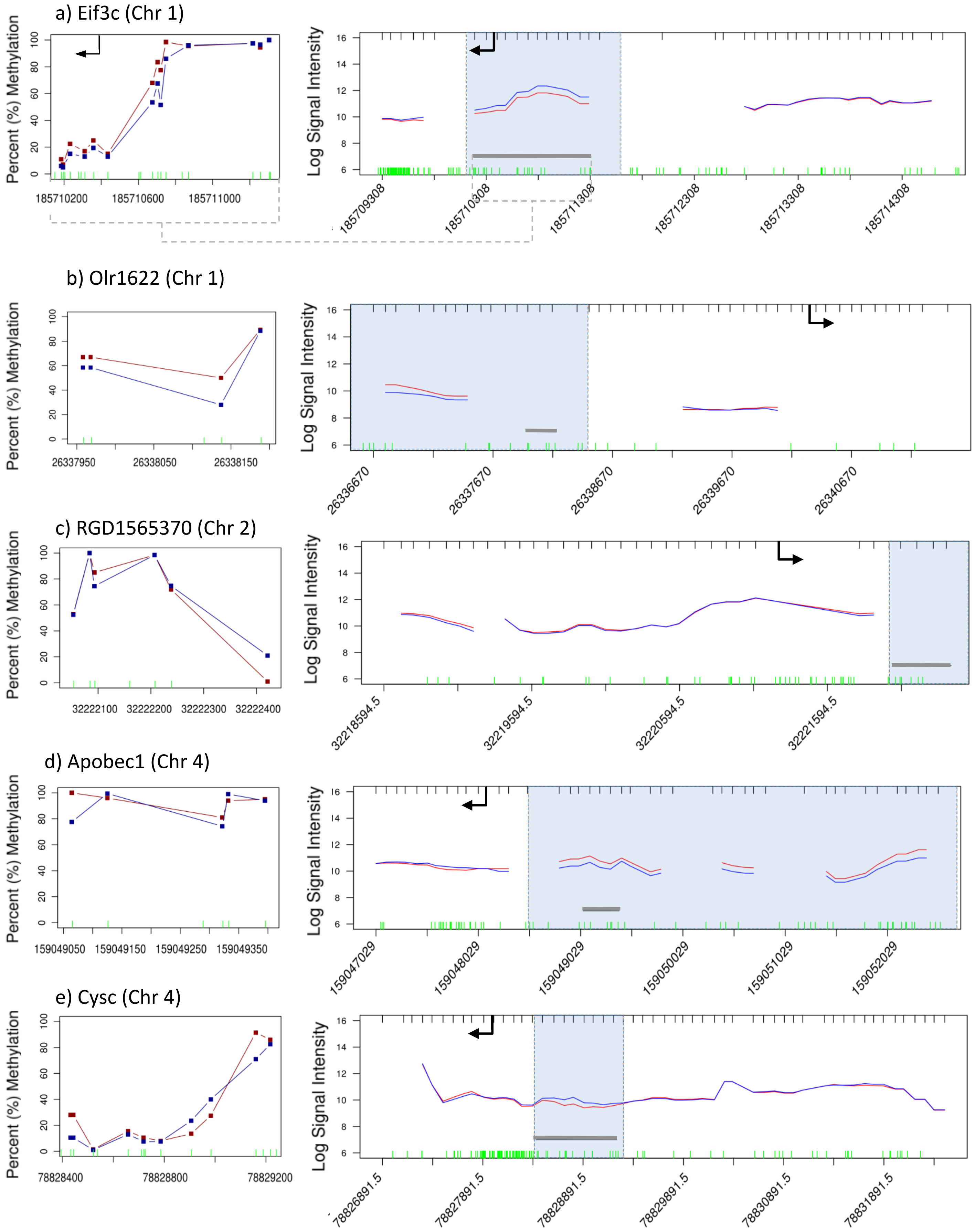

Supplementary Figure S1 (continuation)

f) RGD1359202 (Chr 6)

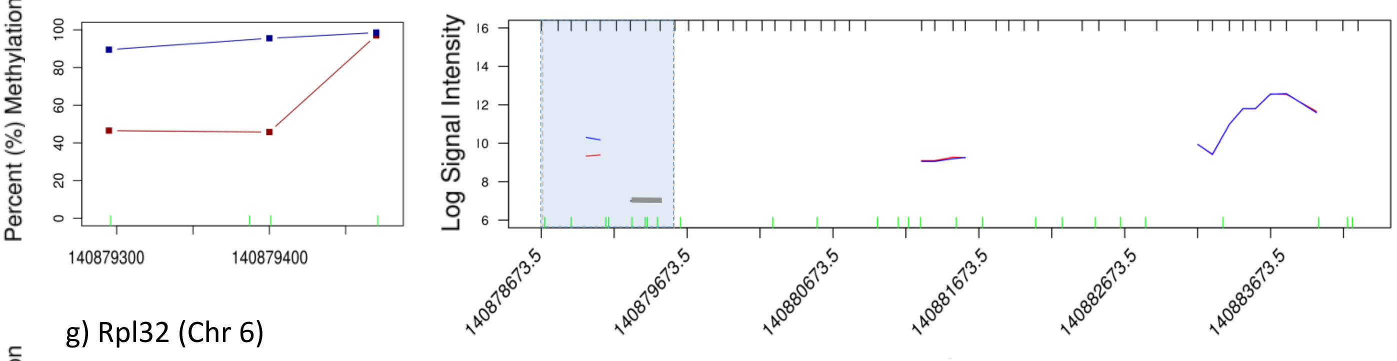

g) Rpl32 (Chr 6)

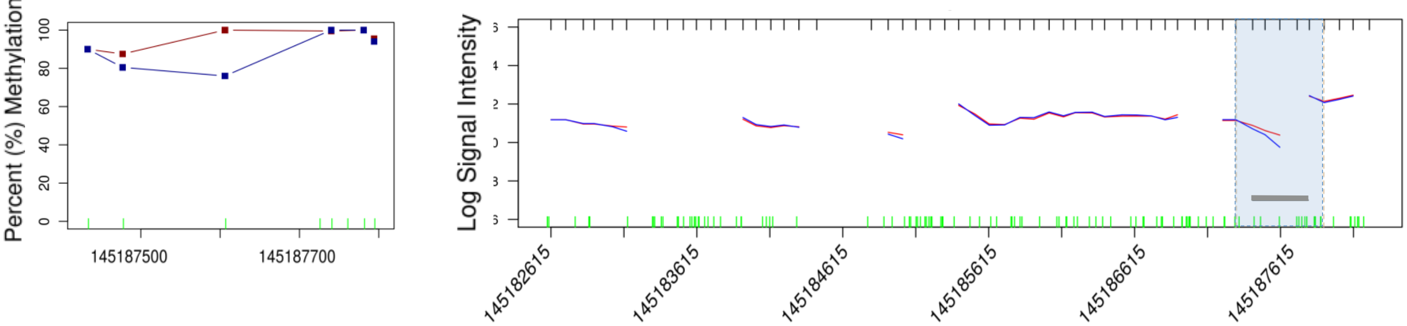

h) LOC688807 (Chr 7)

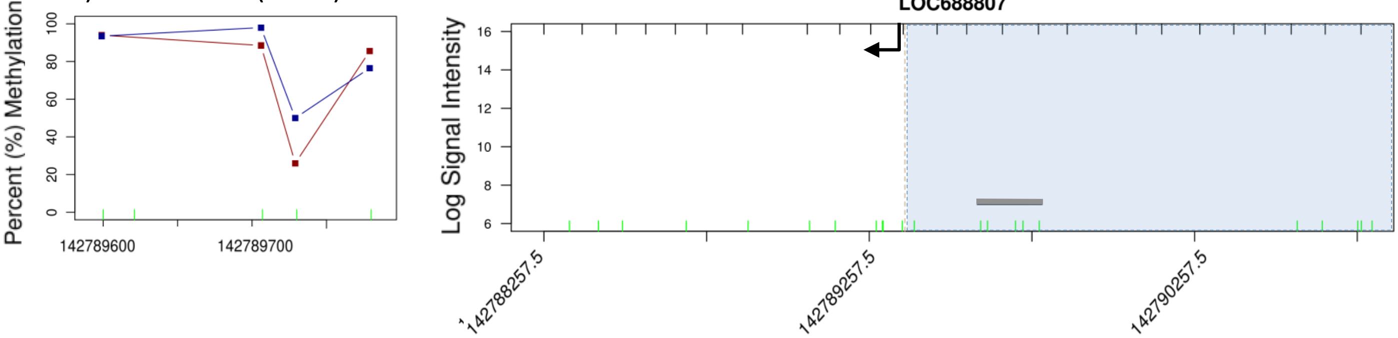

i) LOC690666 (Chr 10)

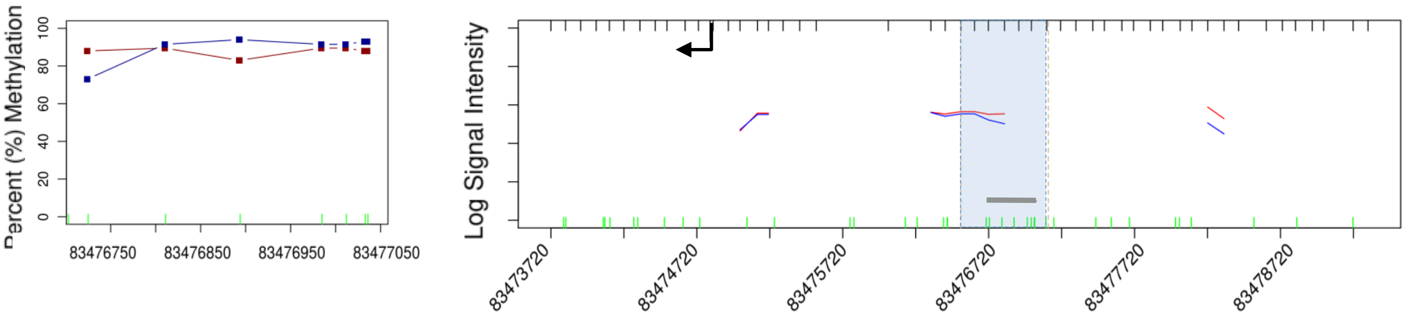

j) Olr129 (Chr 11)

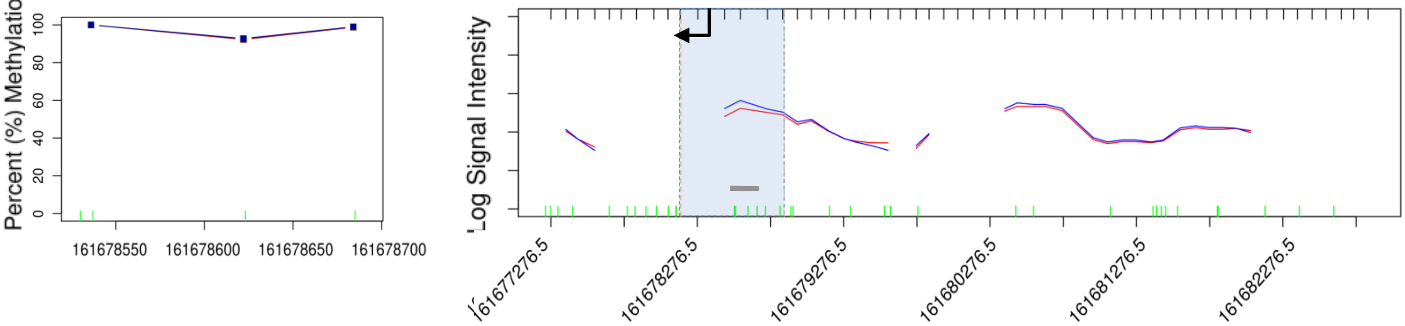

Supplementary Figure S1 (continuation)

k) Olr1549 (Chr 11)

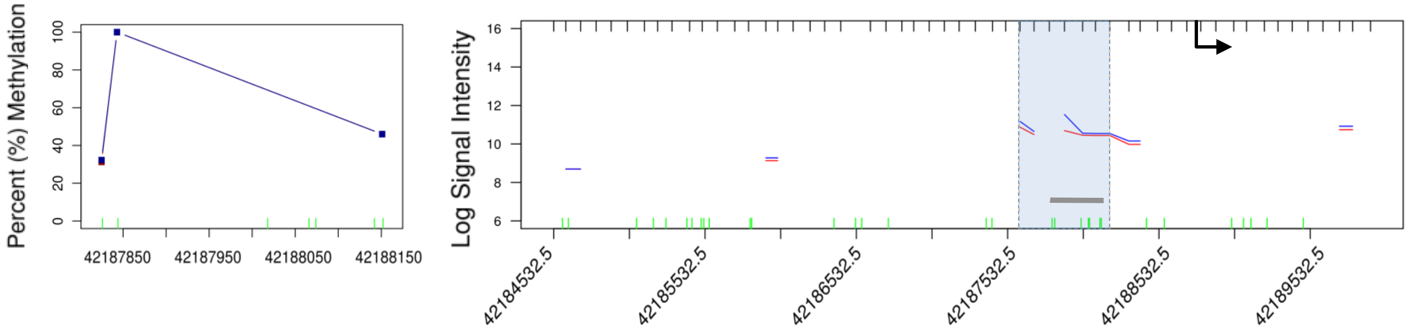

l) Pbx1 (Chr 13)

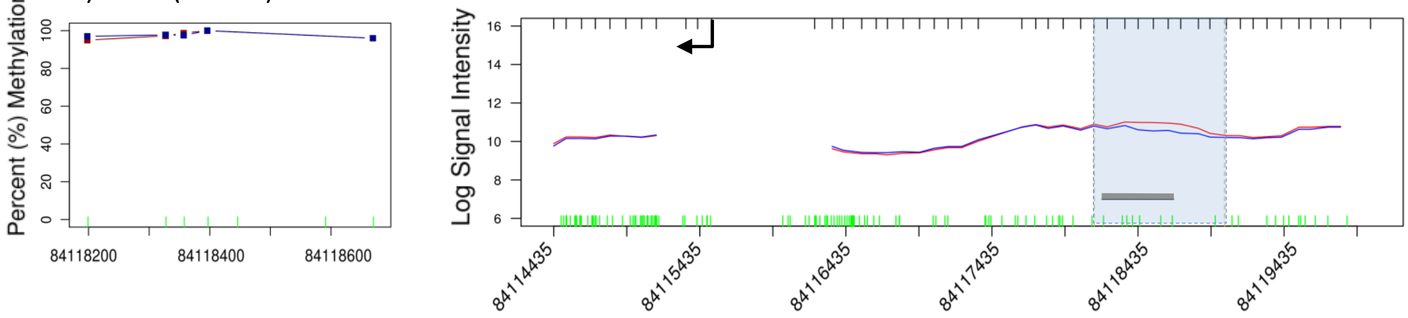

m) Plek (Chr 14)

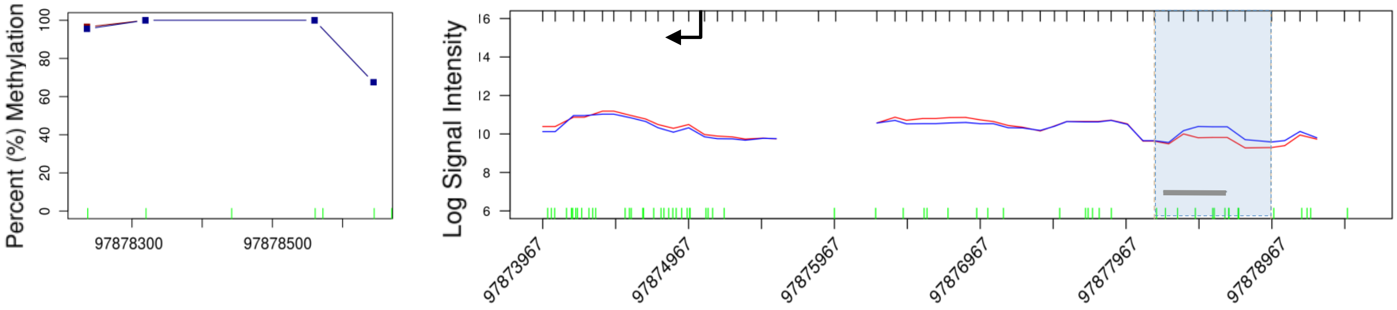

n) B3gnt2 (Chr 14)

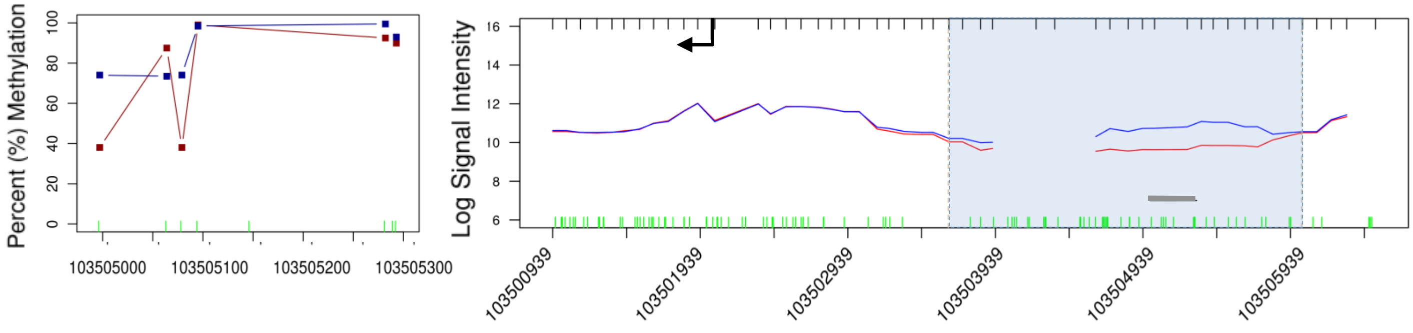

Supplementary Figure S1 (continuation)

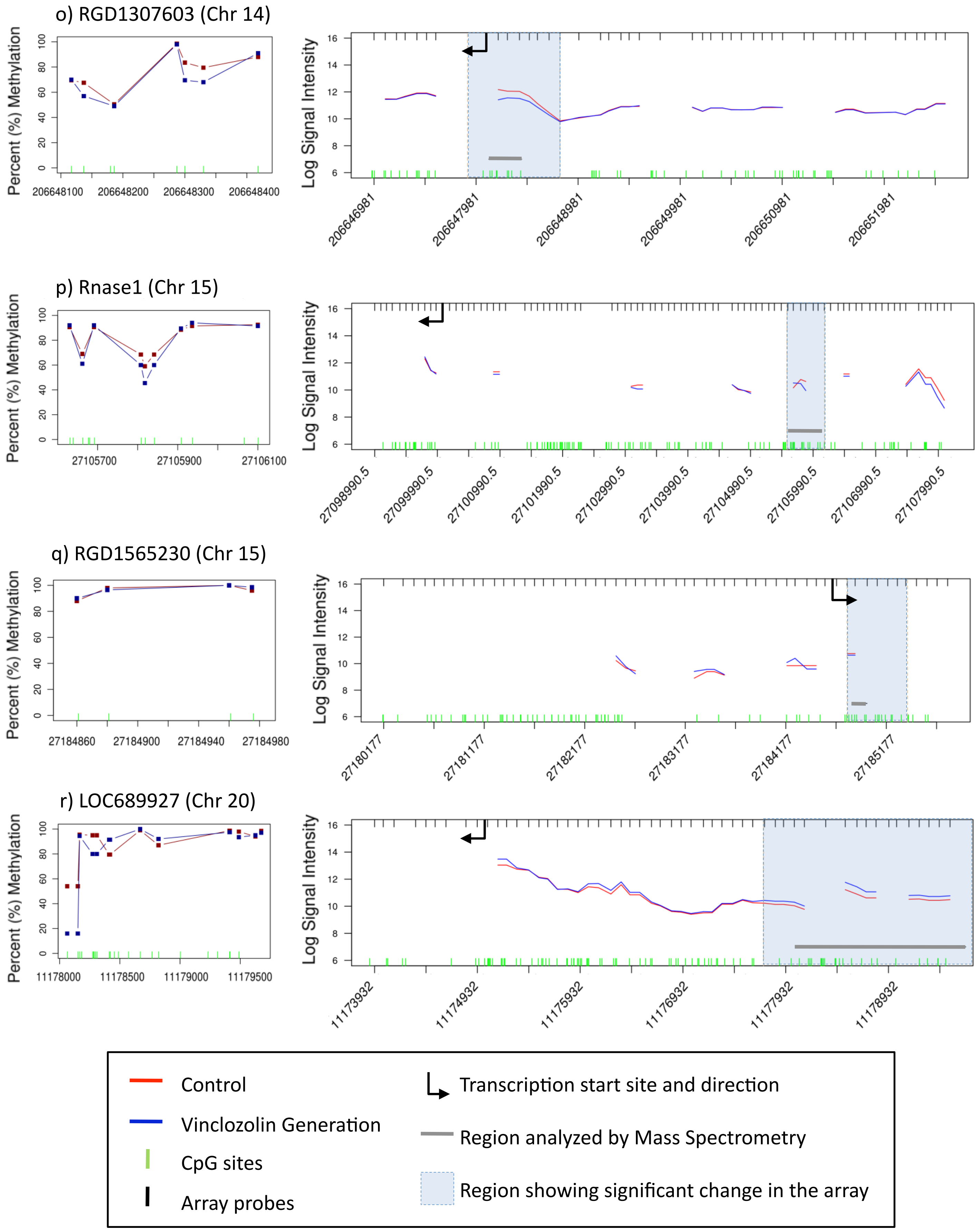

Supplementary Figure S1 – Comparison of the methylation signal in regions where transgenerational methylation change could not be confirmed between vinclozolin and control F3 generation sperm. Analysis of methylation through MeDIP followed by comparative hybridization (right graph) and through bisulfite mass spectrometry (left graph) is shown for each gene **(a-r)**. Horizontal axis shows chromosomal localizations. For the (c) and (h) genes the probe density for hybridization signal was insufficient to allow a tiling graph to be generated in the shaded regions.
