# Supplementary material for: Epigenetic Transgenerational Actions of Vinclozolin on Promoter Regions of the Sperm Epigenome
Source: PLoS One. 2010 Sep 30;5(9):e13100. doi: 10.1371/journal.pone.0013100 (PMC2948035; doi:10.1371/journal.pone.0013100)
Supplement: Table S1 — List of 52 regions (belonging to 48 promoters) showing transgenerational methylation change in the array and their characteristics. Confirmation status, gene name (Rat Genome Database, RGD), known description of function, RGD and Entrez identification numbers, raw p values and chromosome localizations are listed. (0.09 MB PDF) [file pone.0013100.s002.pdf]

Supplementary Table S1

Confirmation status	Gene Name RGD	Description	RGD ID	Entrez ID	Region changed (bp)	Significance (p value ≤)	Chr	Changed region coordinates Start	End
Confirmed	LOC685544	Hypotetical protein	1592604	685544	2005	1.71E-40	chr1	202535485	202537490
Confirmed	LOC685117	Hypothetical protein LOC685117	1597765	685117	714	1.22E-24	chr4	77481458	77482172
Confirmed	RGD1561412/ Olr735	Similar to olfactory receptor Olfr735	1561412	498490	1795	3.16E-23	chr15	26056221	26058016
Confirmed	KCNE2	Potassium voltage-gated channel, Isk-related subfamily, gene 2	621383	171138	1300	4.64E-16	chr11	32282558	32283858
Confirmed	Anxa1	Annexin A1	2118	25380	1215	3.75E-14	chr1	223496856	223498071
Confirmed	Btbd16	BTB (POZ) domain containing 16	1311454	361658	1080	3.98E-12	chr1	190092281	190093361
Confirmed	Parp9	Poly (ADP-ribose) polymerase family, member 9	1307534	303905	1180	5.52E-12	chr11	66646569	66647749
Confirmed	GPR33	Glycoprotein A33 (transmembrane)	1306830	299007	1674	5.62E-11	chr6	72176041	72177715
Confirmed	Eef1d	Eukaryotic translation elongation factor 1 delta (guanine nucleotide exchange protein)	621174	300033	600	1.32E-10	chr7	113878277	113878877
Confirmed	Olr1624	Olfactory receptor 1624	1334059	290001	1485	2.44E-10	chr15	26395012	26396497
Confirmed	RGD1560076/ 1562802	Similar to 60S ribosomal protein L29(P23)	1560076/ 1562802	294722/ 502505	803	2.44E-10	chr2	39456073	39456876
Confirmed	RGD1562552	Similar to hypothetical protein LOC340061	1562552	498840	1495	3.34E-10	chr18	28356310	28357805
Confirmed	Prr13	Prolin rich 13	1307129	363004	1077	1.39E-08	chr7	141214859	141215936
Confirmed	RGD1311451/ Nmral1	NmrA-like family domain containing 1	1311451	287063	1104	2.46E-08	chr10	10967516	10968620
Confirmed	Kcng1	Potassium voltage-gated channel, subfamily G, member 1	631416	296395	1000	6.17E-08	chr3	159439013	159440013
Confirmed	LOC689162	Similar to thyroid hormone receptor associated protein 3	1596142	689162	1995	7.72E-08	chr10	23193568	23195563
Not Confirmed	Fam111a	Family with sequence similarity 111, member A	1560913	499322	4689	3.84E-95	chr1	215587367	215592056
Not Confirmed	B3gnt2	UDP-GlcNAc:betaGal beta-1,3-N-acetylglucosaminyltransfe rase 2	1310077	305571	1695	1.56E-43	chr14	103504320	103506015
Not Confirmed	RGD1307603	Similar to hypothetical protein MGC37914	1307603	293656	905	3.10E-17	chr1	206647898	206648803
Not Confirmed	RGD1359202	Immunoglobulin heavy chain 6	1359202	299357	902	6.57E-17	chr6	140878682	140879584
Not Confirmed	Olr1622	Olfactory receptor 1622	1334259	405127	600	5.65E-14	chr15	26337866	26338466
Not Confirmed	LOC689927	Similar to keratin associated protein 10-10	1586373	689927	2115	9.20E-14	chr20	11177716	11179831
Not Confirmed	Rnase1	Ribonuclease, RNase A family, 1 (pancreatic)	3574	364304	600	1.92E-12	chr15	27105574	27106174
Not Confirmed	Plek	Pleckstrin	1308269	364206	800	1.86E-11	chr14	97878161	97878961
Not Confirmed	RGD1565230	Ribonuclease, RNase A family, 1-like 2 (pancreatic)	1565230	305844	600	2.21E-11	chr15	27184787	27185387
Not Confirmed	Apobec1	Apolipoprotein B mRNA editing enzyme, catalytic polypeptide 1	2133	25383	885	8.33E-11	chr4	159051824	159052709
Not Confirmed	Olr1622	Olfactory receptor 1622	1334259	405127	687	1.29E-10	chr15	26336470	26337157
Not Confirmed	Apobec1	Apolipoprotein B mRNA editing enzyme, catalytic polypeptide 1	2133	25383	996	5.24E-10	chr4	159048520	159049516
Not Confirmed	Eif3c	Eukaryotic translation initiation factor 3, subunit C	1308871	293484	1482	5.62E-09	chr1	185710113	185711595
Not Confirmed	Rpl32	Ribosomal protein L32	621203	28298	600	6.20E-09	chr6	145187311	145187911
Not Confirmed	Cysc	Cystatin C	2432	25307	888	7.72E-09	chr4	78828404	78829292
Not Confirmed	Olr1549	Olfactory receptor 1549	1333231	288196	600	1.05E-08	chr11	42187608	42188208
Not Confirmed	Pbx1	Pre-B-cell leukemia	1308213	304947	892	3.02E-08	chr13	84118136	84119028

		homeobox 1							
Not Confirmed	LOC690666	Transmembrane protein 92	1593405	690666	600	3.78E-08	chr10	83476529	83477129
Not Confirmed	Olr129	Olfactory receptor 129	1332980	405912	706	3.94E-08	chr1	161678164	161678870
Not Confirmed	LOC688807	Hypothetical protein LOC688807	1584159	688807	600	7.51E-08	chr7	142789367	142789967
Not Confirmed	RGD1565370	Similar to ribosomal protein L21	1565370	294700	600	7.87E-08	chr2	32222011	32222611
Not Tested	NSCAN.chr6.1007.a	Predicted gene	NA	NA	1281	1.54E-54	chr6	140861594	140862875
Not Tested	Scgb2a1	Secretoglobin, family 2A, member 1	3424	25010	674	1.77E-17	chr1	212159981	212160655
Not Tested	RGD1561143	Similar to cell surface receptor FDFACT	1561143	304382	1200	3.10E-15	chr12	19995272	19996472
Not Tested	LOC685117	Hypothetical protein LOC685117	1597765	685117	600	3.43E-14	chr4	77478979	77479579
Not Tested	Ighg	Immunoglobulin heavy chain (gamma polypeptide)	1359539	299354	600	5.71E-13	chr6	139089240	139089840
Not Tested	LOC680818	Hypothetical protein LOC680818	1587065	680818	680	5.35E-12	chr6	9041555	9042235
Not Tested	LOC688807	Hypothetical protein LOC688807	1584159	688807	600	3.02E-11	chr7	142790359	142790959
Not Tested	LOC688800	Aldehyde dehydrogenase 3 family, member B2	1584166	688800	600	1.80E-10	chr1	206549843	206550443
Not Tested	St3gal1	ST3 beta-galactoside alpha-2,3-sialyltransferase 1	1309373	362924	903	6.06E-10	chr7	104477658	104478561
Not Tested	RGD1560158	NADH dehydrogenase (ubiquinone) 1 alpha subcomplex, assembly factor 2	1560158	361894	1210	8.58E-10	chr2	39379401	39380611
Not Tested	LOC680077	Hypothetical protein LOC680077	1596811	680077	1797	8.60E-09	chr18	66984394	66986191
Not Tested	Mad2l1	MAD2 (mitotic arrest deficient, homolog)-like 1 (yeast)	1310889	297176	600	8.84E-09	chr4	96379284	96379884
Not Tested	Olr1079	Olfactory receptor 1079	1333801	404960	600	2.45E-08	chr7	12131801	12132401
Not Tested	RGD1562462	Similar to Ifi204 protein	1562462	289245	694	3.11E-08	chr13	89657903	89658597
Not Tested	Ptma	Prothymosin alpha	61829	29222	600	5.22E-08	chr9	85291094	85291694

Supplementary Table S1 – List of 52 regions (belonging to 48 promoters) showing transgenerational methylation change in the array and their characteristics. Confirmation status, gene name (Rat Genome Database, RGD), known description of function, RGD and Entrez identification numbers, raw p values and chromosome localizations are listed.
