# Supplementary material for: Epigenetic Transgenerational Actions of Vinclozolin on Promoter Regions of the Sperm Epigenome
Source: PLoS One. 2010 Sep 30;5(9):e13100. doi: 10.1371/journal.pone.0013100 (PMC2948035; doi:10.1371/journal.pone.0013100)
Supplement: Table S2 — List of amplicons used for mass spectrometry methylation analysis, with information on length, number of CpG sites analyzed, location and strand amplified. (0.96 MB PDF) [file pone.0013100.s003.pdf]

Supplementary Table S2

Anxa1_02	418	7	Chr1:223497026-223497443	(-)
Anxa1_03	425	5	Chr1:223497416-223497840	(-)
B3gnt2_03	410	8	Chr14:103504944-103505353	(+)
chr1.Eif3c_15870	424	9	Chr1:185710157-185710580	(+)
chr1.Eif3c_15871	372	8	Chr1:185710555-185710926	(+)
chr1.Eif3c_15872	365	4	Chr1:185711159 - 185711523	(+)
chr1.Olr129_15856	499	5	Chr1:161678279-161678777	(+)
chr1.Olr129_15867	472	5	Chr1:161678342 - 161678814	(+)
chr1.RGD1307603_15885	169	4	Chr1:206648050 - 206648218	(+)
chr1.RGD1307603_15886	452	4	Chr1:206648226 - 206648677	(+)
chr10.LOC689162_15897	464	7	Chr10:23193662-23194125	(+)
chr10.LOC689162_15898	474	11	Chr10:23194025-23194498	(+)
chr10.LOC689162_15900	433	9	Chr10:23194474-23194906	(+)
chr10.LOC689162_15901	487	13	Chr10:23194881-23195367	(+)
chr10.LOC689162_15902	407	11	Chr10:23195132-23195538	(+)
chr10.LOC690666_15895	486	8	Chr10:83476576 - 83477061	(+)
chr10.RGD1311451_15876	378	5	Chr1:10967642-10968019	(+)
chr10.RGD1311451_15892	417	6	Chr10:10967594 - 10968010	(+)
chr10.RGD1311451_15893	442	8	Chr10:10967988 - 10968429	(+)
chr10.RGD1311451_15894	447	8	Chr10:10968085 - 10968531	(+)
chr11.Olr1549_15903	431	7	Chr11:42187758 - 42188188	(+)
chr11.Parp9_15904	477	8	Chr11:66646681 - 66647157	(+)
chr11.Parp9_15905	269	12	Chr11:66647133 - 66647401	(+)
chr13.Pbx1_15914	446	5	Chr13:84118137-84118582	(+)
chr13.Pbx1_15915	435	6	Chr13:84118274-84118708	(+)
chr14.B3gnt2_15919	307	5	Chr14:103504745-103505051	(+)
chr14.B3gnt2_15921	405	5	Chr3:103505328-103505732	(+)
chr14.Plek_15916	480	6	Chr14:97878189-97878668	(+)
chr15.Olr1622_16008	425	5	Chr15:26337882 -26338306	(+)
chr15.OLR1624_15931	500	9	Chr15:26395116-26395615	(+)
chr15.OLR1624_15932	350	8	Chr15:26395569-26395918	(+)
chr15.OLR1624_15933	466	8	Chr15:26395876-26396341	(+)
chr15.RGD1565230_15926	233	4	Chr15:27184833-27185065	(+)
chr15.Rnase1_15924	305	9	Chr15:27105600-27105904	(+)
chr15.Rnase1_15925	289	4	Chr15:27105879-27106167	(+)
chr2.RGD1565370_15940	318	6	Chr2:32222022-32222339	(+)
chr2.RGD1565370_15941	487	5	Chr2:32222122-32222608	(+)
chr20.LOC689927_15947	490	13	Chr20:11178033-11178522	(+)
chr20.LOC689927_15948	490	4	Chr20:11178576-11179065	(+)

chr20.LOC689927_15949	468	5	Chr20:11178977-11179444	(+)
chr20.LOC689927_15950	492	7	Chr20:11179247-11179738	(+)
chr3.Kcng1_15951	332	7	Chr3:159439016-159439347	(+)
chr3.Kcng1_15952	363	8	Chr3:159439233-159439595	(+)
chr3.Kcng1_15953	413	7	Chr3:159439558-159439970	(+)
chr4.Apobec1_16047	393	6	Chr4:159048968-159049360	(+)
chr4.Apobec1_16048	269	4	Chr4:159049174-159049442	(+)
chr4.Cysc_16044	435	9	Chr4:78828407-78828841	(+)
chr4.Cysc_16045	475	6	Chr4:78828817-78829291	(+)
chr4.LOC685117_16043	287	5	Chr4:77481873-77482159	(+)
chr6.GPR33_16053	447	4	Chr6:72176261-72176707	(+)
chr6.GPR33_16054	481	16	Chr6:72176698-72177178	(+)
chr6.GPR33_16055	497	7	Chr6:72177125-72177621	(+)
chr6.RGD1359202_16050	460	5	Chr6:140878968-140879427	(+)
chr6.RGD1359202_16051	343	4	Chr6:140879237-140879579	(+)
chr6.Rpl32_16052	500	8	Chr6:145187343-145187842	(+)
chr7.Eef1d_16058	428	25	Chr7:113878279-113878706	(+)
chr7.LOC688807_16059	368	5	Chr7:142789508-142789875	(+)
Fam111a_01	490	7	Chr1:215587502-215587991	(+)
Fam111a_04	447	4	Chr1:215588163-215588609	(-)
Fam111a_05	484	4	Chr1:215590419-215590902	(-)
KCNE2_01	390	8	Chr11:32282686-32283075	(+)
KCNE2_03	462	18	Chr11:32283067-32283528	(-)
LOC685544_02	429	5	Chr1:202536052-202536480	(+)
LOC685544_03	298	4	Chr1:202536458-202536755	(+)
LOC685544_05	435	4	Chr1:202535497-202535931	(-)
Prr13_03	520	8	Chr7:141215234-141215753	(-)
rat Btbd16_01	256	13	Chr1:190092286-190092541	(+)
rat Btbd16_02	422	11	Chr1:190092490-190092911	(+)
RGD1307603_03	465	8	Chr1:206648091-206648555	(-)
RGD1560076-RGD1562802_02	598	7	Chr2:39455996-39456593	(-)
RGD1561412/Olr735_01	416	9	Chr15:26056678-26057093	(+)
RGD1561412/Olr735_02	475	11	Chr15:26056932-26057406	(-)
RGD1562552_01	464	10	Chr18:28356435-28356898	(+)
RGD1562552_02	482	12	Chr18:28356798-28357279	(+)
RGD1562552_05	471	9	Chr18:28357306-28357776	(-)

Supplementary Table S2 - List of amplicons used for mass spectrometry methylation analysis, with information on length, number of CpG sites analyzed, location and strand amplified
