# Supplementary material for: Epigenetic Transgenerational Actions of Vinclozolin on Promoter Regions of the Sperm Epigenome
Source: PLoS One. 2010 Sep 30;5(9):e13100. doi: 10.1371/journal.pone.0013100 (PMC2948035; doi:10.1371/journal.pone.0013100)
Supplement: Table S3 — Letter probability matrix for EDM1. Numbers indicate the probability of occurrence of the nucleotide for each position in the motif. (0.02 MB PDF) [file pone.0013100.s004.pdf]

| Nucleotide in Motif | EDM1 letter-probability matrix: A | C | G | T |
|---|---|---|---|---|
| 1 | 0.625 | 0.25 | 0.0625 | 0.0625 |
| 2 | 0.75 | 0.1875 | 0 | 0.0625 |
| 3 | 0.6875 | 0.0625 | 0.1875 | 0.0625 |
| 4 | 0.4375 | 0.5625 | 0 | 0 |
| 5 | 0.875 | 0 | 0 | 0.125 |
| 6 | 0.625 | 0 | 0.0625 | 0.3125 |
| 7 | 0.5625 | 0.25 | 0.1875 | 0 |
| 8 | 0.625 | 0 | 0 | 0.375 |
| 9 | 0 | 0.125 | 0.5625 | 0.3125 |
| 10 | 0.625 | 0.25 | 0.0625 | 0.0625 |
| 11 | 1 | 0 | 0 | 0 |
| 12 | 0.75 | 0.0625 | 0 | 0.1875 |
| 13 | 0.75 | 0 | 0.0625 | 0.1875 |
| 14 | 0.5 | 0.4375 | 0.0625 | 0 |
| 15 | 0.75 | 0.25 | 0 | 0 |
| 16 | 0.8125 | 0 | 0 | 0.1875 |
| 17 | 1 | 0 | 0 | 0 |
| 18 | 0.5625 | 0 | 0.125 | 0.3125 |
| 19 | 0.6875 | 0.25 | 0.0625 | 0 |
| 20 | 0.8125 | 0.125 | 0.0625 | 0 |
| 21 | 0.4375 | 0.375 | 0 | 0.1875 |

Supplementary Table S3: Letter probability matrix for EDM1. Numbers indicate the probability of occurrence of the nucleotide for each position in the motif
