# Supplementary material for: Epigenetic Transgenerational Actions of Vinclozolin on Promoter Regions of the Sperm Epigenome
Source: PLoS One. 2010 Sep 30;5(9):e13100. doi: 10.1371/journal.pone.0013100 (PMC2948035; doi:10.1371/journal.pone.0013100)
Supplement: Table S4 — List of promoters of known imprinted genes that were tested against EDM1.The table includes location of the gene, information regarding if the promoter was from Mus musculus or Rattus norvegicus and also information regarding if EDM1 presented at least one hit inside the promoter region of that gene. The list was compiled from the ‘Catalogue of Parent of Origin Effects: Imprinted Genes and Related Effects’ from the University of Otago, New Zealand. The 75 promoter used were selected because there was information on their Transcription Start Site at the NCBI Nucleotide tool and on the direction of their transcription at the NCBI Gene tool. The promoter region of each gene was calculated by adding 5000 bp upstream and 1200 downstream of the Transcription Start Site (ATG) obtained from the NCBI Nucleotide tool. (0.08 MB PDF) [file pone.0013100.s005.pdf]

Supplementary Table S4

Gene name	Location	Species	At least one region similar to EDM1 with Glam2scan (cut-off score value >20)
Ampd3	Chr7:117911118-117917318	Mus musculus	no
Asb4	Chr6:5328286-5334586	Mus musculus	yes
Ascl2	Chr7:150160132-150153932	Mus musculus	yes
Calcr	Chr6:3719713-3713513	Mus musculus	yes
Cd81	Chr7:150233700-150239900	Mus musculus	yes
Cdkn1c	Chr7:150651901-150645701	Mus musculus	no
Commd1	Chr11:22887284-22881084	Mus musculus	yes
Copg2	Chr6:30851760-30845560	Mus musculus	yes
Ddc	Chr11:11795404-11789204	Mus musculus	yes
Dhcr7	Chr7:151004072-151010272	Mus musculus	no
Dio3	Chr12:111512440-111518640	Mus musculus	no
Dlk1	Chr12:110686433-110692633	Mus musculus	yes
Gatm	Chr2:122441994-122435794	Mus musculus	no
Gnas	Chr2:174104821-174111021	Mus musculus	no
Grb10	Chr11:11942358-11936158	Mus musculus	yes
Hm13_H13	Chr2:152490235-152496435	Mus musculus	yes
Htr2a	Chr14:75035647-75041847	Mus musculus	yes
Igf2	Chr7:149850394-149844194	Mus musculus	yes
Igf2	chr1:202914032-202920230	Rattus norvegicus	no
Igf2r	Chr17:12967572-12961372	Mus musculus	yes
Igf2r	chr1:42248273-42254473	Rattus norvegicus	yes
Impact	Chr18:13125761-13131961	Mus musculus	yes
Impact	chr18:669197-675397	Rattus norvegicus	yes
Ins2	Chr7:149870013-149863813	Mus musculus	no
Kcnk9	Chr15:72381709-72375509	Mus musculus	no
Kcnq1	Chr7:150288159-150294359	Mus musculus	no
Klf14	Chr6:30913982-30907782	Mus musculus	no
MAGEL2	Chr7:69516865-69523065	Mus musculus	no
Mcts2	Chr2:152507884-152514084	Mus musculus	no
Meg8_Rian	Chr12:107119072-107125272	Mus musculus	no
Mest	Chr6:30683063-30689263	Mus musculus	yes
miR-134	Chr12:67720581-67726781	Mus musculus	yes
miR-154	Chr12:67724875-67731075	Mus musculus	yes
miR-380	Chr12:67698245-67704445	Mus musculus	yes
miR-410	Chr12:67730157-67736357	Mus musculus	no
miR-411	Chr12:67696617-67702817	Mus musculus	yes
Mirg	Chr12:110963996-110970196	Mus musculus	no
Mirn184	Chr9:9363503-9357303	Mus musculus	no
Mirn335	Chr6:27686309-27692509	Mus musculus	yes
Mkrn3	Chr7:69570025-69563825	Mus musculus	no
Nap1L4	Chr7:150739995-150733795	Mus musculus	yes
Nap1l5	Chr6:58862059-58855859	Mus musculus	yes
Ndn	Chr7:69488163-69494363	Mus musculus	yes
Nnat	Chr2:157380846-157387046	Mus musculus	yes

Osbpl5	Chr7:150932868-150926668	Mus musculus	no
Peg10	Chr6:4692306-4698506	Mus musculus	yes
Peg12	Chr7:69614396-69608196	Mus musculus	yes
Peg3	Chr7:6688130-6681930	Mus musculus	no
Phlda2	Chr7:150693429-150687229	Mus musculus	yes
Plagl1	Chr10:12805715-12811915	Mus musculus	yes
Ppp1r9a	Chr6:4848320-4854520	Mus musculus	yes
Rasgrf1	Chr9:89799613-89805813	Mus musculus	no
Rasgrf1	chr8:94797543-94803743	Rattus norvegicus	yes
Rtl1	Chr12:110838613-110832413	Mus musculus	yes
Sfmbt2	Chr2:10289209-10295409	Mus musculus	yes
Sgce	Chr6:4702099-4695899	Mus musculus	yes
Slc22a18	Chr7:150654660-150660860	Mus musculus	no
Slc22a2	Chr17:12772055-12778255	Mus musculus	no
Slc22a3	Chr17:12705570-12699370	Mus musculus	no
Slc38a4	Chr15:96891387-96885187	Mus musculus	yes
Snrpn	Chr7:67290105-67283905	Mus musculus	yes
Snurf	Chr7:67155042-67148842	Mus musculus	no
Th	Chr7:150090871-150084671	Mus musculus	no
Tnfrsf23	Chr7:150876777-150870577	Mus musculus	yes
Tspan32	Chr7:150186596-150192796	Mus musculus	yes
Tssc4	Chr7:150250273-150256473	Mus musculus	yes
Ube3a	Chr7:66479120-66485320	Mus musculus	yes
Usp29	Chr7:6678452-6684652	Mus musculus	no
Xlrb3b	chrX:70,432,518-70,438,718	Mus musculus	yes
Xlr4b	ChrX:70454704-70460904	Mus musculus	yes
Xlr4c	ChrX:70493469-70487269	Mus musculus	no
Zim1	Chr7:6654143-6647943	Mus musculus	no
Zim2	Chr7:6620117-6613917	Mus musculus	no
Zfp264	Chr7:3701698-3707898	Mus musculus	no
Zrsr1	Chr11:22867029-22873229	Mus musculus	yes

Supplementary Table S4 - List of promoters of known imprinted genes that were tested against EDM1.The table includes location of the gene, information regarding if the promoter was from *Mus musculus* or *Rattus norvegicus* and also information regarding if EDM1 presented at least one hit inside the promoter region of that gene.
The list was compiled from the 'Catalogue of Parent of Origin Effects: Imprinted Genes and Related Effects' from the University of Otago, New Zealand. The 75 promoter used were selected because there was information on their Transcription Start Site at the NCBI Nucleotide tool and on the direction of their transcription at the NCBI Gene tool. The promoter region of each gene was calculated by adding 5000 bp upstream and 1200 downstream of the Transcription Start Site (ATG) obtained from the NCBI Nucleotide tool.
